# Defining the mobility range of a hinge-type connection using molecular dynamics and metadynamics

**DOI:** 10.1371/journal.pone.0230962

**Published:** 2020-04-13

**Authors:** Philip Horx, Armin Geyer

**Affiliations:** Department of Chemistry, Philipps-Universität Marburg, Marburg, Germany; Universidade Nova de Lisboa Instituto de Tecnologia Quimica e Biologica, PORTUGAL

## Abstract

A designed disulfide-rich β-hairpin peptide that dimerizes spontaneously served as a hinge-type connection between proteins. Here, we analyze the range of dynamics of this hinge dimer with the aim of proposing new applications for the DNA-encodable peptide and establishing guidelines for the computational analysis of other disulfide hinges. A recent structural analysis based on nuclear magnetic resonance spectroscopy and ion mobility spectrometry revealed an averaged conformation in the hinge region which motivated us to investigate the dynamic behavior using a combination of molecular dynamics simulation, metadynamics and free energy surface analysis to characterize the conformational space available to the hinge. Principal component analysis uncovered two slow modes of the peptide, namely, the opening and closing motion and twisting of the two β-hairpins assembling the hinge. Applying a collective variable (CV) that mimics the first dominating mode, led to a major expansion of the conformational space. The description of the dynamics could be achieved by analysis of the opening angle and the twisting of the β-hairpins and, thus, offers a methodology that can also be transferred to other derivatives. It has been demonstrated that the hinge peptide’s lowest energy conformation consists of a large opening angle and strong twist but is separated by small energy barriers and can, thus, adopt a closed and untwisted structure. With the aim of proposing further applications for the hinge peptide, we simulated its behavior in the sterically congested environment of a four-helix bundle. Preliminary investigations show that one helix is pushed out and a three-helix bundle forms. The insights gained into the dynamics of the tetra-disulfide peptide and analytical guidelines developed in this study may contribute to the understanding of the structure and function of more complex hinge-type proteins, such as the IgG antibody family.

## Introduction

Covalent bonding between identical proteins can fulfill numerous purposes depending on the flexibility of the linker. The unstructured highly flexible linker of a Lys-ε-amide bond in ubiquitylation [1,2] allows for the free rotation of the linked domains, while the highly ordered contact surface in dimeric defensin covalently fixed by three intermolecular disulfide bonds prevents any independent mobility of either domain [3]. In both cases, the structural integrity and the resulting dynamics can exert a decisive influence on the biological functions of the proteins [4,5]. Hinge domains are in between these extremes, allowing restricted relative movements and, therefore, resembling an interesting target for modeling techniques [6]. A hinge, as the name already implies, can be characterized by various mechanical attributes, such as the minimal vs. maximal opening angle and its opening-closing frequency. The most prominent example is the bi-disulfide hinge of IgG1 antibodies, whose directed mobility is a key component for its specific activity against numerous pathogens and, thus, the development of monoclonal antibodies has been a rapidly expanding field in the last few decades [7–10]. This mobility is composed of several conformational changes, varying from rotation of a single bond of one residue to complex conformational rearrangements, which have a major impact of the shape of the protein [11]. The likelihood of these conformational changes depends on their energetic properties, which can be projected onto a free energy surface. A combination of experimental and computational methods can be used to investigate this dynamic. Structural elucidation can be performed with crystallography [12] and nuclear magnetic resonance (NMR) spectroscopy [13] and yield starting structures which often already resemble the lowest energy structure. For a long time, structural biology has focused on low-energy structures, while recently, the dynamic behavior of proteins has been increasingly appreciated [14,15]. Nevertheless, the low-energy structure serves as a good starting point for dynamic studies using various spectroscopic methods or molecular dynamics simulation. Molecular dynamics (MD) simulation a particularly useful tool for gaining insight into the free energy surface, which, as explained above, can assist in determining pathways for biological processes [16]. The time scale for such processes is in the range of milliseconds or even longer, which means that for an accurate determination, the resulting trajectory has to sample the conformational space extensively, generating a need for enhanced sampling methods. These methods can range from replica exchange MD [17] to steered MD [18] or metadynamics [19]. The latter combines several properties of other methods, including the reduction of dimensions and the ability to reconstruct the free energy surfaces [20]. Metadynamics can be visualized as filling energy basins with computational sand (a positive Gaussian potential), which allows the sampling of other minima and, thus, covering the conformational landscape. This method has been employed and modified for the investigation of a vast amount of different scientific questions since its development in 2002 [21–26]. In a recent study, [27] bovine pancreatic trypsin inhibitor was used as a model system for the determination of a small number of slow collective variables which could be used for the identification of metastable states of the disulfide-rich protein.

We recently described a new type of tetradisulfide hinge which forms by dimerization of a 12mer tetra-Cys peptide [28,29]. The high regioselectivity of disulfide formation allows its application as a dimerization hinge with the aim of producing covalent protein homodimers. The NMR spectroscopy and ion-mobility mass spectrometric measurements yielded time-averaged data suggesting an intrinsic dynamic of the peptide hinge compromising an opening and closing motion which led us to entitle the compound a “hinge peptide” and to use it successfully as a hinge domain in a monoclonal antibody. In this study, we performed classical molecular dynamics simulation in conjunction with well-tempered metadynamics to get a deeper understanding of the hinge peptide’s dynamics. Principal component analysis (PCA) aided in uncovering functionally relevant collective motions [30] On the basis of these motions, we proposed a single collective variable (CV), that is readily transferable to other dimeric hinge derivatives, to increase sampling of the free energy surface. This data set is intended to support the search for new applications of tetra-disulfide hinges, for which we describe one example in the final chapter. In addition, the approach described here can serve as a reference for the analysis of other hinge-type large-scale protein motions.

## Results

### β-hairpin structure

**Fig 1:**
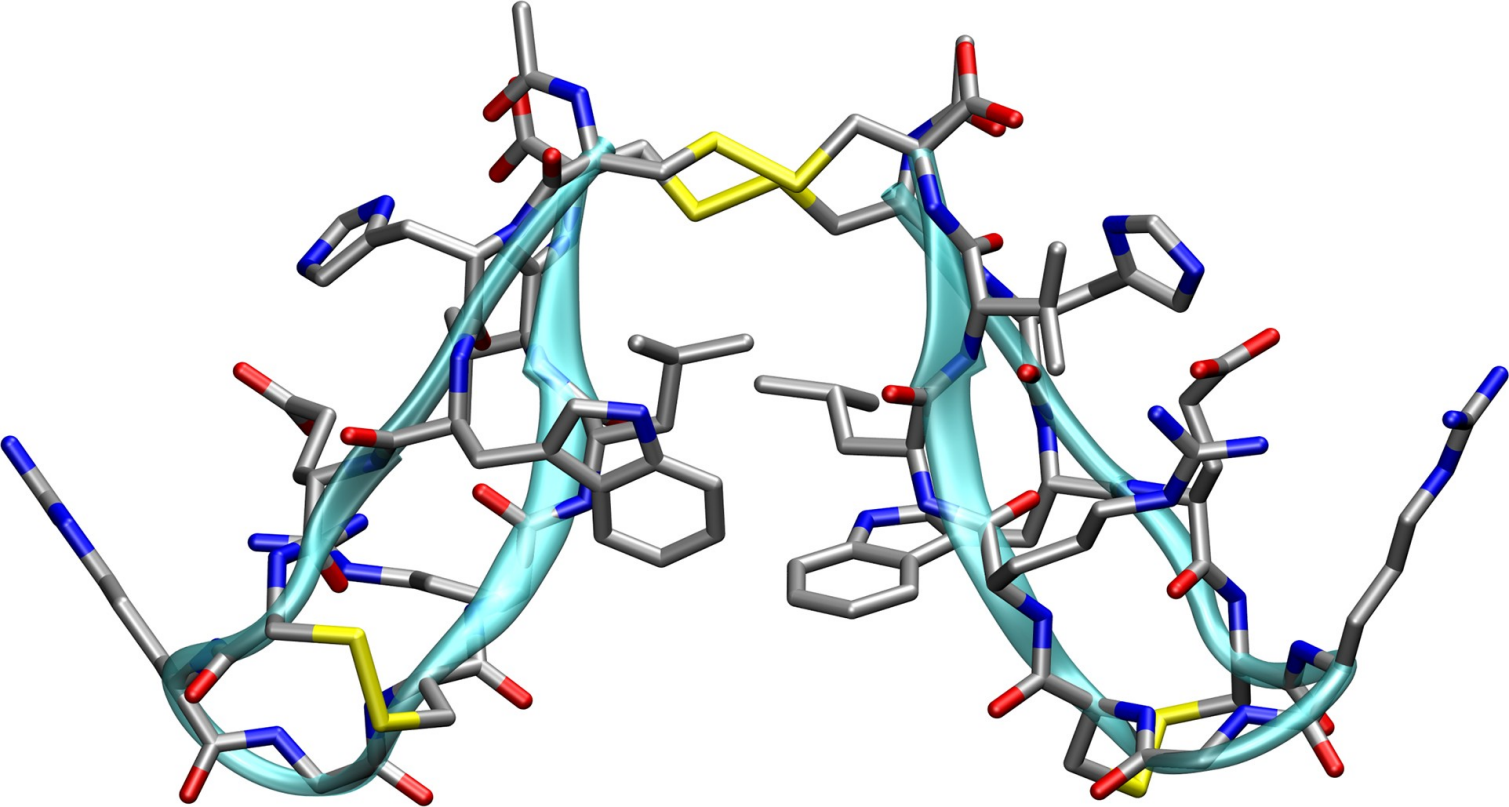
Starting structure of the dimeric hinge peptide. Lowest energy conformer of the dimeric hinge peptide (CHWECRGCRLVC)_2_ obtained after a simulated annealing protocol using an NMR-derived nuclear Overhauser effect and dihedral restraints. The hydrogen atoms are omitted for better visibility.

NMR analysis of the C2-symmetric C1-C12’,C1’-C12, C5-C8, C5’-C8’ tetradisulfide peptide yielded geometric restraints for a preliminary energy-minimized structure, as shown in [28]. To further increase the applicability of the tetradisulfide peptide for biochemical purposes, the sequence was modified to a DNA-encodable 12mer sequence CHWECRGCRLVC, which was successfully used to dimerize the prokaryotic enzyme limonene epoxide hydrolase [29]. Following the NMR structure protocol, which is explained in detail in the Computational Details section, the same twisted β-hairpin structure could be observed, with slight variations in some side-chain orientations. Even though the overall twist is a characteristic feature of standalone β-hairpins [31,32], the 12mer β-hairpin of the title compound cannot form a consistent number of hydrogen-bonded and non-bonded pairs, which is characteristic for an antiparallel β-sheet, and is further restricted in the β-turn region due to the C-5-C8 (C5’-C8’, respectively) intramolecular disulfide, which is visible in the NMR-derived structure in Fig 1. The combination of both effects prevents the formation of the macrocyclic C1-C12 disulfide of a single β-hairpin but promotes the oxidation to the intermolecular C1-C12’ (C1’-C12, respectively) dimer. This claim is further supported by the absence of a detectable amount of monomeric bi-disulfide β-hairpin, whose structure is not formed during the oxidation. The small number of fluctuations obtained in the final NMR ensemble are only located in the side chain residues, which serves as an indicator of the stability of the hinge peptide.

### Uncovering hidden dynamics of the hinge peptide

The NMR-derived structure served as the starting point for unbiased molecular dynamics simulation, which was performed to gain insight into the stability and motions the hinge peptide exhibits at room temperature. Visual inspection of the 2500ns long trajectory showed several conformational changes but local small scale fluctuations obscure the large-scale collective motions. Slow and sometimes correlated motions can play a key role in the function of proteins and enzymes [33]. To overcome this challenge, dimension reduction techniques are routinely used, to elucidate large-scale and low-frequency modes “hidden” in the multidimensional conformational space. One established method, which is routinely applied not only to peptides and proteins but other multidimensional data sets in general, is PCA [34,35]. We constructed the covariance-matrix using the Cα-atoms of each chain and projected the trajectory on the resulting eigenvectors. The first eigenvector, which accounts for 55% of the covariance, describes a twisting motion of the β-hairpin motifs to each other, bringing the aliphatic sidechains W3 and L10 in close contact. A hinge-like opening and closing dynamics could be observed on the second eigenvector (11% of the covariance). The distribution of the first 10 Eigenvectors are reported in the Supporting Information.

### Selecting collective variables for enhanced sampling

After identification of rare events from unbiased MD simulations, metadynamics was performed to enhance sampling and to overcome energy barriers, thus characterizing the hinge peptides’ dynamics. One challenge within the metadynamics methodology is the choice of a collective variable(s). While it is possible to use the eigenvectors obtained from the PCA as a suitable choice for the enhanced sampling, it is desirable to select a geometric parameter, which is applicable to other dimeric hinge peptides. Thus, the free energy plots of the isolated hinge peptide, the hinge peptide with attached protein loads or mutants of the hinge peptide become more comparable. Therefore two CVs were defined accordingly. First, the opening angle, determined by the midpoints between the Cα-atoms of both intramolecular disulfides and the center of the intermolecular disulfide cluster, was chosen as the collective variable. While this obvious choice leads to a reasonable enhanced sampling of the conformational space sampled (SI), a neglected degree of freedom is the twisting of the β-hairpin motifs. To overcome this challenge, we identified the distance between the center of the indole groups as a single CV. We observed that by biasing this variable both, the twisting of the β-hairpin motifs and the opening and closing of the hinge were facilitated, which led to an increase in maximum and minimum values, while retaining the secondary structure of the hinge peptide, determined by the number of hydrogen bonds during the simulation. A combination of several CVs (around three) is frequently chosen to bias a simulation [36]. Naturally, the combination of both CVs was investigated but led to an unfolding of the secondary structure at a certain point during the simulation. By tuning the height and biasfactor, conditions could be found, under which the secondary structures were preserved. Comparison with the W–W’ distance bias showed that the conformational area sampled is roughly the same, but free energy calculations revealed that the system samples only one area. It can, therefore, be concluded that the W-W’ distance bias as the single CV is feasible for enhanced sampling to aid in the understanding of the hinge peptides’ dynamics.

### Analysis of the hinge peptide’s dynamics

**Fig 2:**
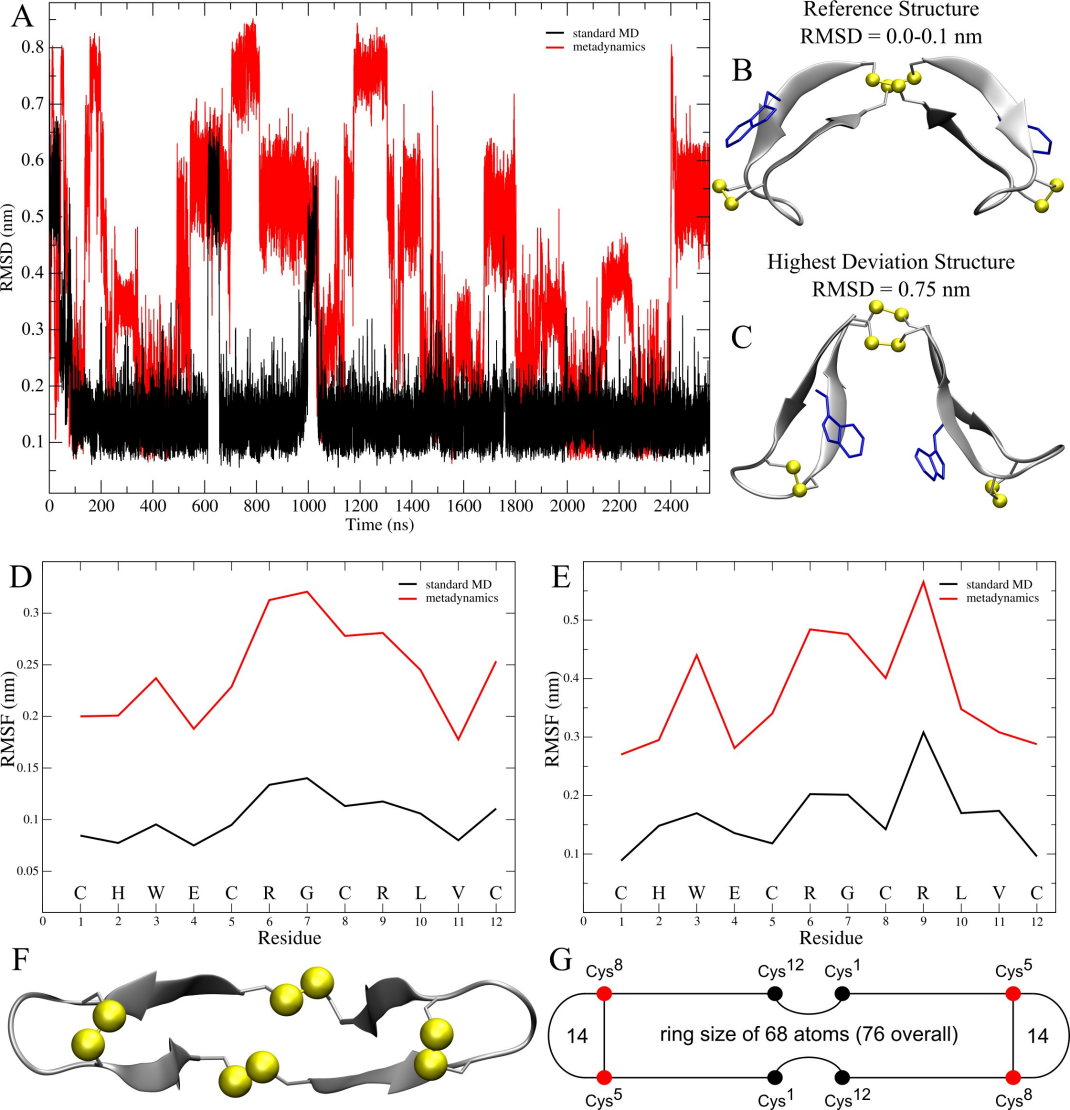
Root-mean-squared Deviation (RMSD) and root-mean-square fluctuation (RMSF) analysis of the hinge peptide with representation of important structural features. The RMSD value development during the classical (MD) simulation (black) and metadynamics (red) (A). On the right, structural representation of the reference structure (B) and highest deviation structure in comparison to the reference (C) during the MD simulation with their respective RMSD value. The RMSF of the backbone atoms (D) and side chain residues (E) are depicted. Bottom view of the hinge peptide backbone with cysteine as bridgehead atoms (F) next to the simplification of these structural features (G).

The routinely employed method of root-mean-squared deviation (RMSD) was used to evaluate the general stability or conformational changes of the hinge peptide. It serves as an indicator of conformational stability in the system during the simulation. As shown in Fig 2(A), the RMSD value in the classical MD simulation, after initial fluctuations during the first 100 ns, converges to a value of 0.15 nm, with only small jumps during the simulation indicating that the hinge peptide equilibrated to a different conformation. The last frame of the MD simulation, which was chosen as the reference structure (B) shows a large twist of the β-hairpins which forces the tryptophan-side chains in an orientation away from each other. During the simulation, a higher RMSD value of 0.55 nm is reached briefly, which indicates structural change closer to the starting structure. By comparison, the use of metadynamics forces the structure to visit different states regularly, which is visible during the whole simulation time frame. Furthermore, the structural changes are more severe, since higher RMSD values are reached, indicating that new regions are explored. The conformation showing the highest RMSD value (0.75 nm) is depicted in Fig 2(C). Root-mean square fluctuation (RMSF) can measure how much the position of a specific residue varies around the average structure to further describe the extent of conformational flexibility of protein and peptide derivatives [37]. We performed RMSF analysis on the backbone and side chains seperately and depicted the results, averaged over the two chains, in Fig 2D and 2E. Analysing the backbone, the loop region R6 and G7 shows a higher fluctuation than the rest of the hinge peptide, which is a characteristic for most β-hairpin structures [38]. In detail, R6 and G7 display a RMSF value of 0.15 nm, while the other residues range from 0.06–0.13 nm. The RMSF pattern is largely retained in the biased simulation. Interestingly, RMSF analysis with a focus on the side chains reveals a different fluctuation pattern. Minimal values of 0.1 nm are observed this time at the terminal cysteine residues C1 and C12, which correspond to the well-defined geometry of the disulfide cluster in the hinge motif. On the other hand, the intra-strand disulfides (C5-C8;C5’-C8’) exhibit a slightly higher RMSF value (0.13–0.15 nm), further supporting the rigidity of the disulfide cluster whose pattern is almost completely retained in the metadynamics simulation. Since R9 does not form a salt bridge with E4 and the C-terminus like R6, it shows the largest side chain fluctuation. By biasing the W–W’ distance, the pattern remains identical varying only in E4 and V11. This hints that the peptide indeed acts like a hinge, in which the anchor point remains largely stable (Fig 2G). Furthermore, while the backbone fluctuation of R9 (0.28 nm) in the metadynamics remains rather low, the side chain value increases to the highest of the system with 0.55 nm.

### Characterizing the hinge type motion

**Fig 3:**
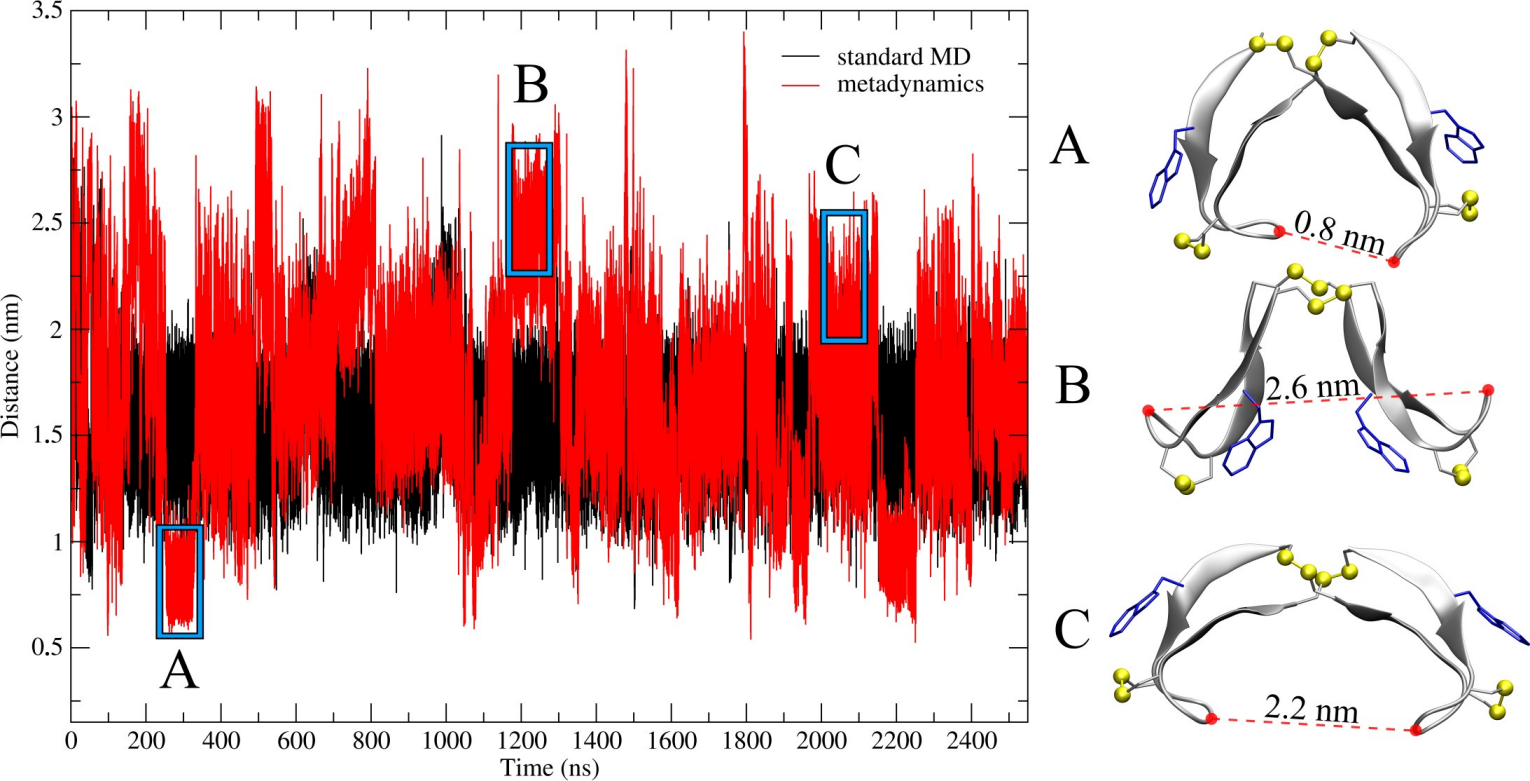
Hinge motion constituted as loop region distance. Representation of the opening and closing motion by development of the distance between the α-carbons of G7 and G7’ located in the turn region. The time-dependent evolution during the standard MD simulation in black and using metadynamics in red. The highlighted regions resemble closest distance (A), large distance (B) and medium distance (C) with a large opening angle.

While RMSD and RMSF pose as a nice tool for the overall structural analysis of proteins and peptides, it would be one-sided to restrict the analysis to these methods. Therefore, we looked for an alternative to describe the hinge-type motion of the peptide. One discernible choice is the distance of both loop regions in the β-hairpin from each other, since this would describe the opening and closing motion. Measurement of the G7 and G7’ α-carbon atom distance situated in the turn region revealed a course similar to the RMSD value. Fig 3 shows the evolution of the distance during the MD simulation and stays consistent with the Fig 2 –i.e. the system rarely undergoes changes in its structure. The distance equilibrates at 1.5 nm with small jumps to 2 nm at 700 and 1000 ns, which depicts neither an open nor closed conformation,

This behavior changes drastically in the well-tempered (wt)-metadynamics simulation. We observed a distinct fluctuations of distance, indicating an opening and closing motion. Furthermore the hinge peptide is able to sample a wider range of distances. Frequent transitions from completely closed (0.8 nm, Fig 3A) to extended (2.6 nm Fig 3B) can be observed. The loop distance is determined mainly by the opening and closing motion but also by the turn region which itself displays a mobility, causing distance variations without changing the opening angle, as highlighted in Fig 3 area C. Further imprecision arises from the twisting motion, which could orientate the loop region close to another, resulting in a smaller distance, indicating a false conformational transition to a closed structure. In order to overcome this challenge and avoid errors in the determination of the open or closed state of the hinge peptide, a direct measurement of the opening angle, eliminating the error introduced by loop flexibility and β-hairpin twisting, would be beneficial. It is possible to describe much more complex motions and interactions with virtual atoms, as highlighted in the literature [39]. Taking the geometric center between C5α and C8α combined with the center of the four sulfur atoms constituting the disulfide cluster, yields an opening angle which corresponds to the typical hinge motion and eliminates fluctuations of the turn region, thus, yielding the opening and closing modes without influence of the mobility of the loop region.

**Fig 4:**
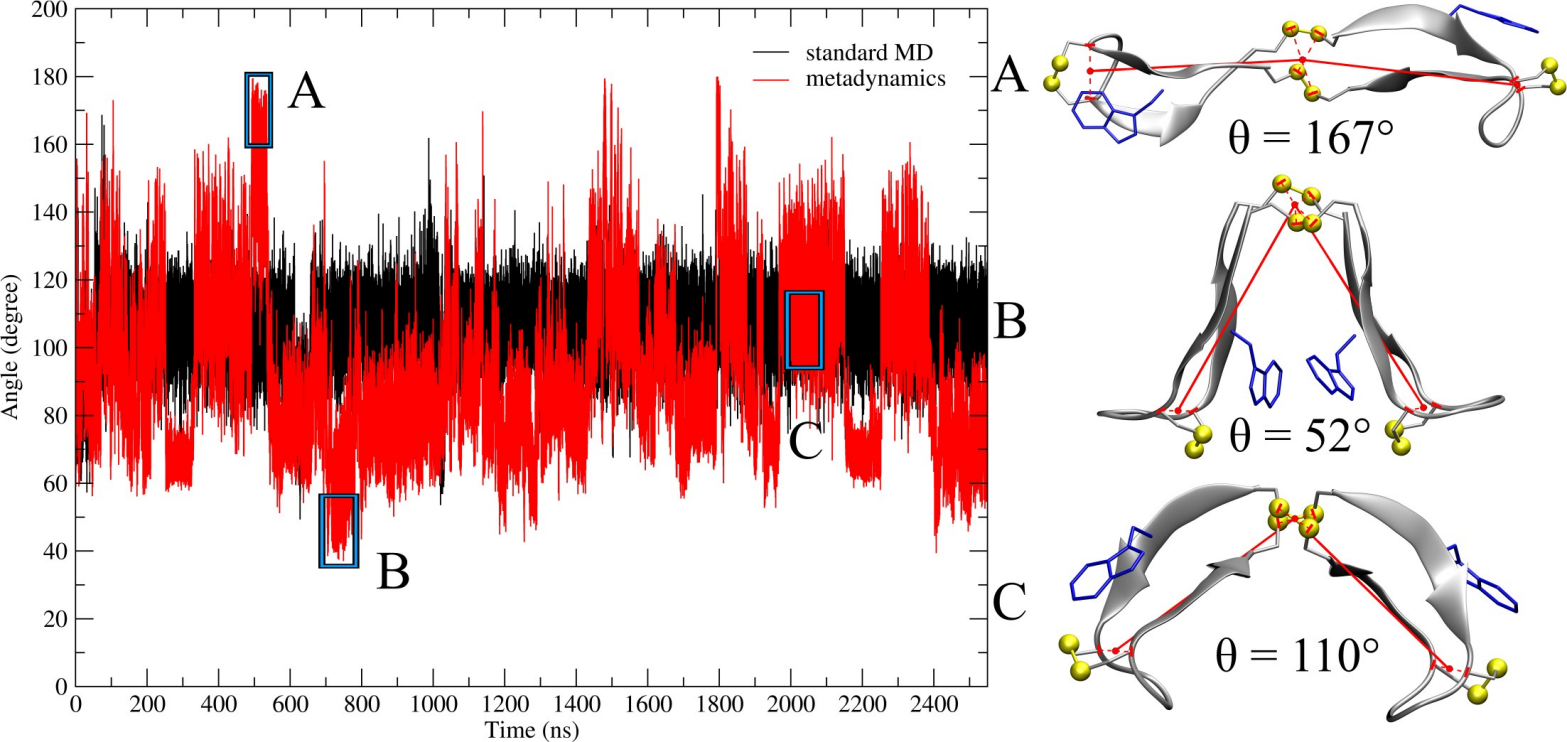
Hinge type motion described by the opening angle. Opening angle representation by taking the geometric center between C5/8 and C5’/8’, respectively. The midpoint was built as the geometric center between the four sulfur atoms forming the disulfide cluster. The opening angle evolution during the standard MD simulation is depicted in black and metadynamics in red. Highlighted area A shows the maximum opening angle, area B, the minimum and area C, an averaged opening angle. The structural representation for each area is presented on the right.

The opening angle during standard MD simulation (Fig 4) shows little change in comparison with Fig 3 and stays at a value of 110°, only dipping twice below 80°. In comparison, wt-metadynamics shows a different pattern. Although the hinge peptide reaches its largest angle of 170° again at 500 ns, the minimum is not situated at 300 ns but rather at 700 ns. This behavior differs drastically to Fig 3, where the loop region distance increases from 1.5 nm to 2.5 nm during 600–800 ns which would correspond to a large opening angle. During this time frame, the actual opening angle reaches its lowest value of 40°, representing the most closed conformation, highlighted in Fig 4B. Fig 4, area C, shows a semi-opened state, where the β-hairpin motifs exhibit a large twist, indicated by the large W–W’ distance. This leads to the conclusion that the description of the opening angle using virtual atoms results in a more accurate distinction between opened and closed conformation.

### Description of the hinge peptide’s conformational space

**Fig 5:**
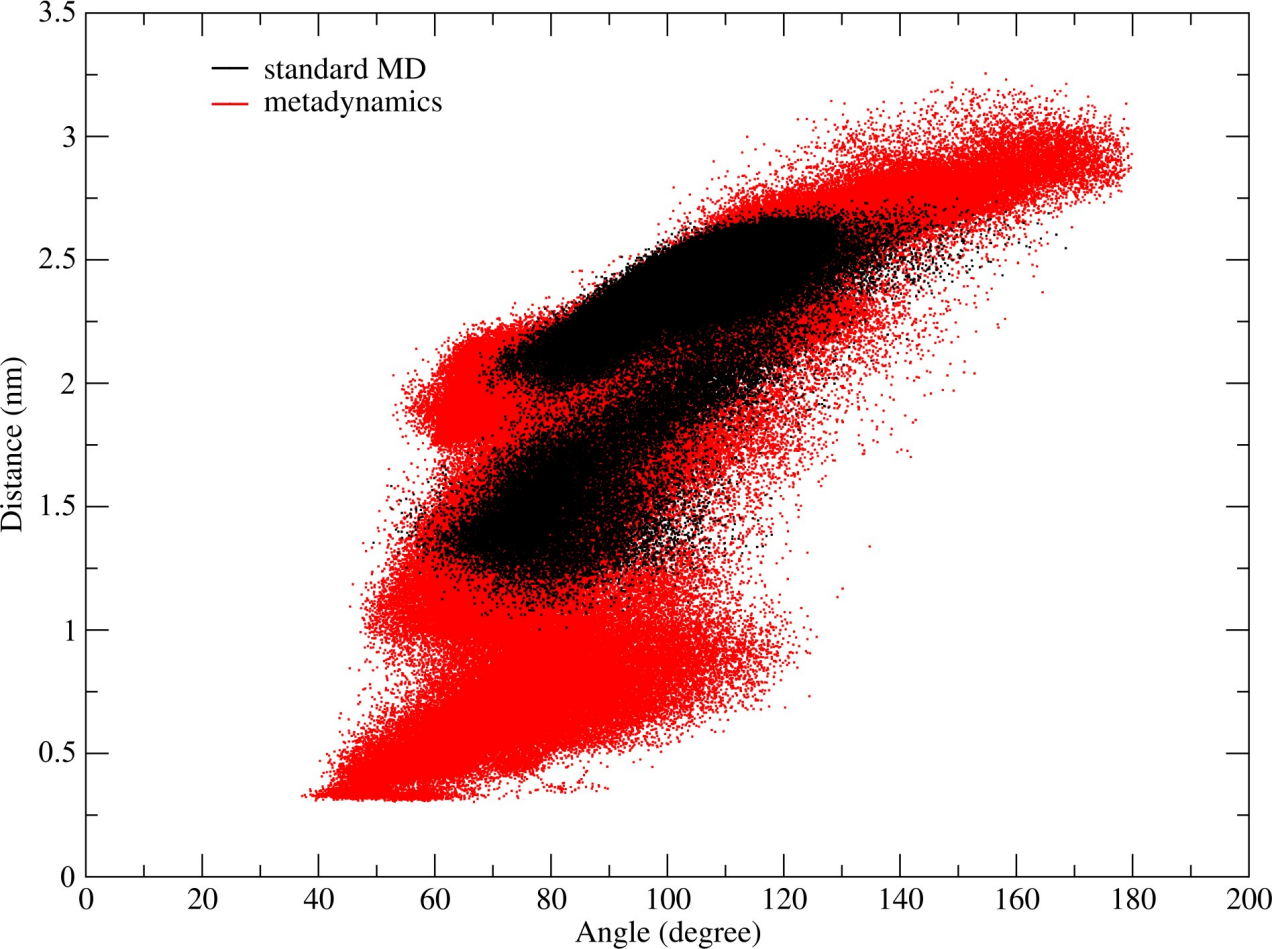
Two-dimensional visualization of the hinge peptide’s sampled conformational space. Plotting the opening angle against the W3 –W3’ distance as a descriptor for twisting of the β-hairpins by taking the geometric center of every tryptophan side-chain atom. The conformational space sampled during standard MD is visualized in black while the metadynamics simulation is shown in red.

While time-dependent analysis of once specific value during a simulation can yield a significant insight into the mechanism for various biological processes, such as inhibitor binding [40], analyzing 2-or 3-dimensional projections can lead to a more concise understanding of conformational dynamics. In the previous chapter, we identified the opening angle constituted by virtual atoms between the cysteine residues as one major contributor to the hinge motion. PCA revealed that the projection of the trajectory on the eigenvector with the highest covariance results in a twisting motion. During this motion the downward facing side chains (V and W) are brought in close contact to their dimeric counterpart. Therefore, to mimic this motion we chose the CV (W–W’ distance) as the second dimension for Fig 5. There were two conformational distinctive areas being visited during the classical MD simulation. While one area shows an opening angle of 75–125° with a narrow W–W’ distance of 2.3 nm. The second group of structures exhibits a smaller opening angle of 70–100°, whith the distance varying between 1.25–2.00 nm. As observed in the 1D-analytical methods, classic MD simulation samples insufficient, while wt-metadynamics further increases the conformational space, which results in three separate regions. Both areas sampled by MD simulation are also sampled during wt-metadynamics, yet distributed over a wider range on the free energy plot. Additionally, a third, previously not observed area of 40–110° and a W–W’ distance of 0.3–1.0 nm is sampled and represents closed, completely twisted conformations.

With the aim of visualizing the energetic properties for each conformational area, free energy calculations were performed by using a weighted histogram approach [41,42]. Since MD simulation only insufficiently samples the available conformational space, only the wt-metadynamics simulation was analyzed.

**Fig 6:**
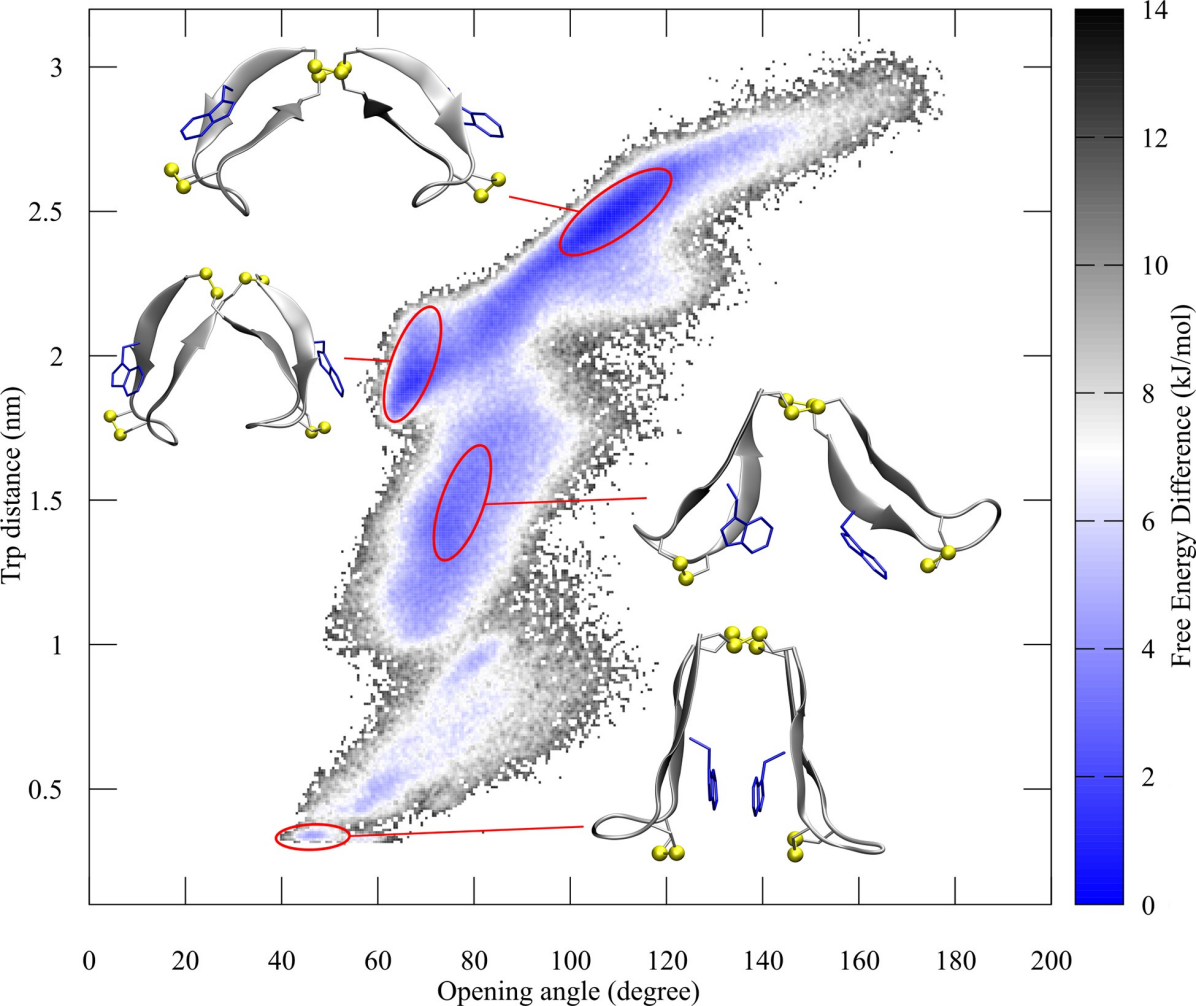
Free energy surface representation of the hinge peptide’s conformational space. Schematic free energy surface of the conformational space built by the opening angle and W–W’ distance. The accentuated areas with their respective structure resemble conformations of interest, ranging from the lowest energy conformation (opened and twisted) at the top to the more unfavored closed and untwisted conformer with a short W–W’ distance. The color scheme represents the energy difference of the various conformations from the reference structure.

FES analysis reveals distinct regions of importance, as depicted in Fig 6. For instance, the area 100–120° opening angle and 2.5 nm distance resembles the conformers with the lowest energy and, thus, depicts the most commonly found conformation in the trajectory. This area represents an almost completely opened conformation with a highly twisted β-hairpin motif. A focus solely on this structure though would not suffice in mapping the dynamics of the hinge peptide, since the second most dominant area lies separated by a small energy barrier of ~4 kJ/mol at 65° and 2 nm, resulting in a closing of the angle while retaining the amount of β-hairpin twist. The largest conformational area that is still energetically favored resolves from 60–100° and 1.00–1.75 nm. In this state, the hinge peptide can undergo an opening and closing motion, whereas the tryptophan residues are facing each other, culminating in a smaller twist value. One well defined area, which is located at 45° and 0.4 nm depicts the hinge in the most closed, non-twisted conformation. Both the opening angle and the twist take minimal values, as the tryptophan residues are in close contact and face each other. Several orientations of the tryptophan residues are possible for this small opening angle, allowing different interactions of both indole groups like π–π stacking. The free energy surface clearly reveals that the hinge peptide is able to undergo an opening and closing motion in combination with the twisting motion to adapt various conformations between closed and opened states.

## Discussion

Ion-mobility spectrometry and NMR spectroscopy yielded time-averaged analytical data and only gave hints regarding the dynamic behavior of the tetradisulfide hinge peptide. Therefore, molecular dynamics simulations were carried out to investigate the structural fluctuation of the system. PCA revealed that the twisting of the β-hairpin motif and the opening and closing of the hinge serve as the most relevant motion in the tetradisulfide peptide. Using standard MD conditions, the system sampled a limited conformational space which led to the exploitation of biased simulation in the form of wt-metadynamics to increase sampling. Various methods for the analysis of the trajectory were employed, starting with simple distance measurements of the loop regions, which gave a first indicator of the opening and closing motion but introduced errors, since its dependency on the internal mobility of the loop region led to incorrect distances. A more successful approach utilized virtual atoms formed by the cysteine residues located in the β-hairpin structure and the center of the disulfide cluster. The opening angle can, thus, be characterized, averaging from 62–85° with smaller values down to 45° resembling a closed structure, and up to 160° fully extended. The second motion, the twisting of the β-hairpin motifs, can be visualized by analyzing the tryptophan distance to each other, since, depending on the opening angle, a twisting of the β-hairpin structure also changes the tryptophan distance. Thus, plotting of the opening angle against the W–W’ distance reveals the conformational space available to the hinge. Naturally, the smallest W–W’ distance of 0.4 nm is only achievable if the hinge is completely closed at 40°. Opening of the structure allows a variety of W–W’ distances up to 2.2 nm, while the longest distance of almost 3 nm is only achieved by a completely extended structure. Free energy calculations using a histogram weighted approach were performed to further investigate the conformational landscape, revealing confined areas separated by small energy barriers. The ensemble with the lowest free energy is located at 60° and 2 nm, which corresponds to a rather closed structure but with highly twisted β-hairpins. The largest area, which stretches from 55–90° and 1.00–1.75 nm, resembles a closed to medium closed structure with different types of twist in the β-hairpins. A rather confined area, which still resembles an energetically favored ensemble is located at 45°/0.4 nm and highlights the most closed structure. Here, the tryptophan residues are in close contact and can engage in face to face or edge to face interactions.

**Fig 7:**
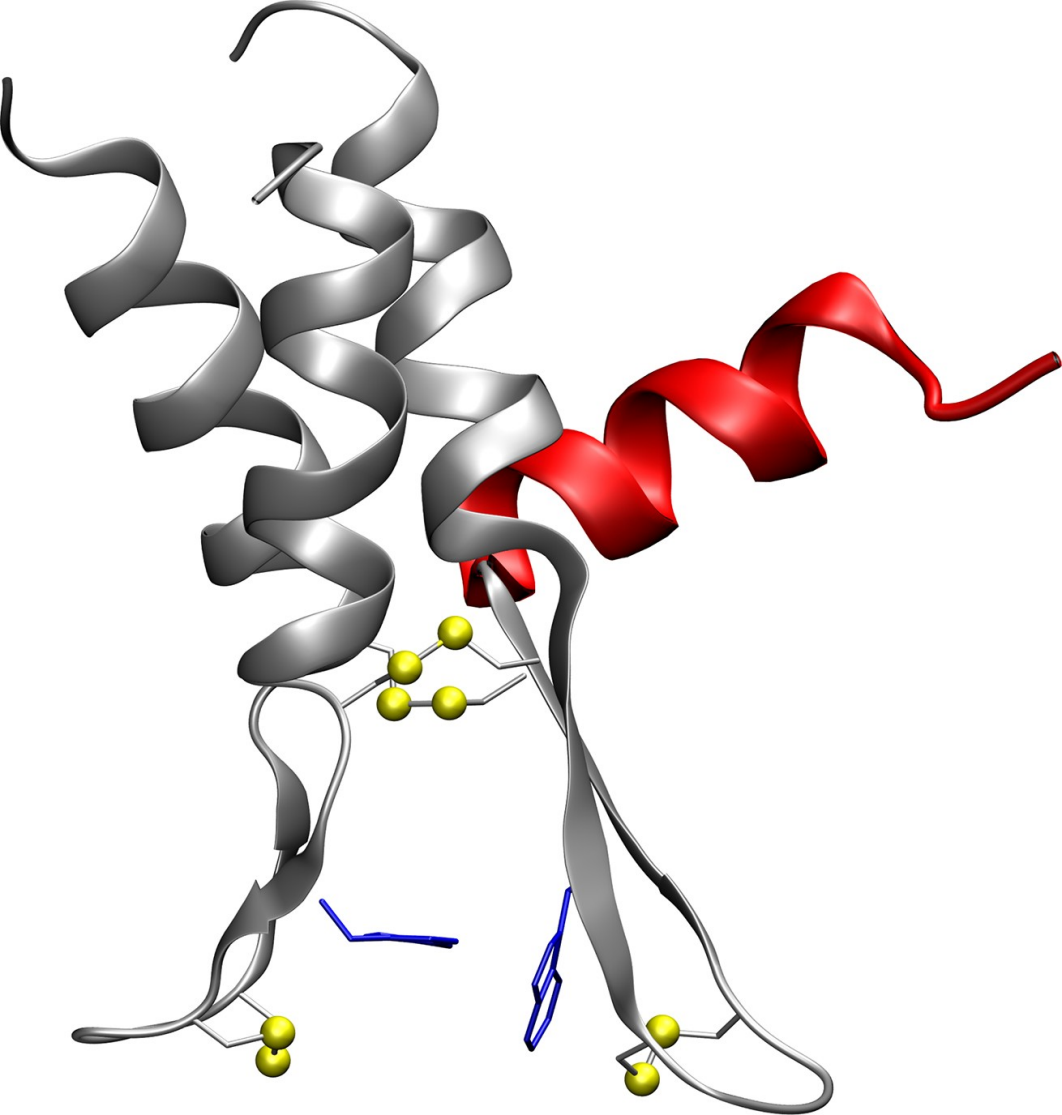
Possible application of the hinge peptide’s restricted dynamics elongated with a 4-helix bundle. Graphical representation of a snapshot taken during the 300-ns-long standard MD simulation of the hinge peptide elongated on the C- and N-terminus by a poly-alanine(14) sequence restricted to an α-helix. Three helices form a bundle, while the fourth one (displayed in red) is directed away due to steric hindrance. This serves as an theoretical example for the unprecedented applications that the dimerization of various elongated hinge peptide sequences can provide.

The 12 amino acids that form the tetra-disulfide hinge can generally be linked N-terminally, internally or C-terminally to additional protein motifs. Therefore, the hinge peptide can be utilized for the dimerization of protein domains. The dynamics described above allow restricted mobility of the attached protein moieties, which can be tuned by inserting glycine or oligo-glycine linker between hinge and protein. In addition, if an internal hinge sequence and the four attached residues come too close, steric interactions can lead to unprecedented results. As an example, we elongated the C- and N-terminus by a 14-residue-long polyalanine α-helix with restraints on its secondary structure yielding the (Ala_14_-CHWECRGCLVC-Ala_14_)_2_ model peptide. The motif of a four-helix bundle was chosen since it is commonly found in several biologically important molecules, such as the HAMP domain [43], ferritin [44] and the ColE1 Rop protein [45]. The helices can be oriented either parallel or antiparallel to each other and their collective motion provides insights into the mechanism of signal transfer in biochemical processes [46]. After a short simulation duration of 300 ns, the structure equilibrates to the conformer shown in Fig 7. The hinge peptide forces three out of four α-helices together, while the fourth helix is unable to stick close due to steric demand.

In conclusion, this study presents analytical guidelines for the computational characterization of the dynamics of a tetradisulfide hinge that was used successfully in previous studies for the dimerization of proteins. By combining MD simulation, PCA and wt-metadynamics the conformational dynamics of the hinge peptide could be uncovered and further elucidated. The dynamics can be described by two dominant motions, namely, the twisting of the β-hairpin motifs and the opening and closing of the hinge. The description of the conformational space using these motions and free energy surface analysis allows the identification of energetically favorable conformations separated by low-energy barriers. This knowledge can be used to investigate the compatibility of the tetradisulfide hinge with other secondary structures, such as helix bundles, and to improve the applications for dimerization of other proteins by the hinge peptide. The in-depth analysis of these dimeric proteins is beyond the scope of this study. However, the insight into the hinge peptide’s dynamics can be exploited to better elucidate the motions for larger hinges, such as those present in the IgG antibody [47].

## Computational details

### Nuclear Overhauser effect restraints

Structure determination of the hinge peptide was performed using the Xplor-NIH suite of programs [48]. Distance constraints were extracted from NOESY spectra with a mixing time of 300 ms. The cross-peaks were divided according to their intensities as weak, medium or strong (2.1–3.1 Å). Due to the flexibility of the system given by size, the focus was shifted on a small set of well-defined distance constraints which would describe the system sufficiently and allows a higher sampling of possible starting structures. This resulted in a total of 23 individual restraints for one chain (46 counting the second strand). Further assessment of coupling constants and cross-peaks revealed preferred side chain orientations which were included as a constraint during the calculation protocol. This data set proved to be consistent in the deliverance of a structural motif with no nuclear Overhauser effect violations above 0.5 Å. The calculations started from an extended conformation using the torsion angle dynamics simulated annealing protocol written by Stein et al. [49]. The starting structure with the sequence (CHWECRGCRLVC)_2_ was acetylated at the N-terminus and deprotonated at the C-terminus to represent the experimental conditions. The sequence started with all four disulfide bonds already in place. The system was heated to 3500 K and cooled down in 12.5 K steps. Instead of the standard Xplor force field, the eefx2 implicit force field was chosen, since it displays higher accuracy and quality of the calculated structure in comparison to the native state [50]. The calculation was performed with 5000 structures while the 20 most stable conformations which showed a large resemblance to each other were extracted. The structure with the lowest energy was chosen as the starting structure for all further molecular dynamics simulations. All structural representations were generated using the VMD graphics suite [51].

### MD simulations

All simulations were performed using the GROMACS 2018.4 [52–54] suite. The lowest energy structure originating from the NMR calculations was chosen for input preparation and processed using the pdb2gmx programm. The dimeric peptide was solvated with ~6000 TIP3P water molecules in a dodecahedron box. A salt concentration of 0.15M was added, in addition to charge-balancing counter ions, to neutralize the system and mimic the environment in which the NMR measurements were originally performed. The relevance of the salt concentration for the stabilization of folded conformations has been outlined in several publications [55,56]. Several force fields (including Amber99SBILDN, CHARMM27, CHARMM36, OPLSAA and OPLSAA/M) were evaluated in short MD simulations at room and elevated temperature. While it has been investigated previously that the OPLSAA force field can fail to adopt a correct β-hairpin fold [57], the improved revision of OPLSAA/M addresses this challenge. The OPLSAA/M force field [58] showed new transitions in the RMSD-value not observed in the other force fields which is in agreement with an averaged structure hinted by in NMR and ion-mobility measurements and, thus, was selected for the modeling. Energy minimization was performed for either 500,000 steps or until the maximum force reached a value below 50 kJ/mol/nm using the steepest-descent algorithm to remove steric clashes between the peptide and solvent. The next step included a 10-ns-ong equilibration to allow the solvent to fully surround the peptide. The first equilibration was conducted under a NVT ensemble at 300 K using the modified Berendsen thermostat v-rescale [59] with a coupling time step of 0.1 ps to stabilize the temperature of the system, followed by an 10-ns-long NPT equilibration to stabilize the pressure using the Berendsen barostat with a coupling time step of 2.0 ps. Particle-Mesh Ewald for the treatment of electrostatic interactions was employed with a short-range cut off of 1.0 nm. The Parrinello-Rahman barostat [60,61] was used for the final unrestrained production run and the system subjected to a 2550-ns-long run at 300K. After the simulation had finished, corrections to the periodic boundary were performed and rotational plus translational motions were removed for easier visualization in the Visual Molecular Dynamics package [51], which was also used to generate the graphical illustrations of the hinge peptide. The RMSD and RMSF were measured using implemented tools in the GROMACS package. A PCA was performed on the whole trajectory of the classical MD simulation to analyze the functionally most relevant motions (more detailed information is presented in the Supporting Information). For the Distance measurement the α-carbon atoms of the Glycine 7 were taken. The geometric center between the α-carbon atoms of Cysteine 5 and 8 and the center of the four sulfur atoms of Cysteine 1 and 12 were taken to define the angle.

### Well-tempered metadynamics

Gromacs 2018.4 patched with plumed 2.4 [62]. was used for wt-metadynamics simulation to increase the sampling of conformational space of the hinge peptide. Several biased parameters (CVs) were evaluated. The geometric centre of the tryptophan residues was chosen as the first CV and let to an increased sampling of the conformational space. The second choice was the opening angle, defined by the geometric centre between the alpha carbons of the intramolecular disulfides and the centre of the intermolecular disulfide cluster. The wt-metadynamics of this CV lead to a limited expansion of the conformational space and thus was unsuitable. Combining both CVs lead to an unfolding of the secondary structure and required lowering of the Gaussian height, which lead to a slower convergence. Thus the W–W’ distance was chosen as the only CV. A Gaussian with a height of 0.2 kJ/mol and a bias factor of 10 was deposited every 1 ps for the well-tempered metadynamics. The Gaussian width was 0.05. These settings were chosen to allow the sampling of the conformational space in a reasonable amount of simulation time, while still allowed to visit smaller energy basins. An increase of the bias factor or Gaussian height yielded an unfolding of the secondary structure. The free energy surface analysis was performed, firstly, by calculating a histogram along the opening angle and W–W’ distance with the kernel density estimation implemented in plumed. Secondly, this data set was then converted to a free energy surface using a weighted histogram approach with a bin size of 425. In order to estimate the conversion of the free energy surface during the simulation, this approach was performed at specific time intervals (after 500, 1000, 2000 and 2550 ns) to evaluate the change in the surface. The corresponding energy surfaces are depicted in the supporting information.

## Supporting information

S1 Text The documents contains the following parts. (DOCX)

S1 Fig Eigenvalues obtained after PCA performed on classical MD simulation. Only the first 10 Eigenvectors are displayed since they account for 82% of the overall motion.
(TIF)

S2 Fig Lowest energy conformer assembly obtained after performing an NMR structure calculation using the simulated annealing protocol. (TIF)

S3 Fig Metadynamics conversion estimation. On the top, the HILLS file generated during the metadynamics simulation shows a constant decline of the deposited Gaussian height. On the bottom, tryptophan distance used as the collective variable shows fluctuations even after no big Gaussian is deployed, signaling a conversion of the simulation.
(TIF)

S4 Fig Free energy surface at different time points. On the top, the graph depicts the FES of the biased parameter during the metadynamics (W–W’ distance) at different time points over the whole simulation. The bottom illustration highlights the resemblance between the free energy profiles at different time steps during the end of the simulation.
(TIF)

S5 Fig Two-dimensional free energy surface evolution. Depiction of the two-dimensional FES at different time points. Large changes can be observed between 500 and 1000 ns but after 2000 ns, the surface changes are only negligibly small. The bottom illustration shows the FES omitting the first 500 ns of the metadynamics simulation.
(TIF)

S6 Fig Free energy estimate profile in combination with error estimation. The graph shows the final FES obtained at the end of the simulation in analogy to S4 Fig. The main basins are labeled and the table on the right shows an estimation of the errors according to each basin.
(TIF)

S7 Fig Free energy surface representations of different metadynamics. A shows the FES of the metadynamics simulation using the tryptophan distance as a CV, while B (opening angle is biased) shows a narrow valley extended in the opening angle but restricted in the trp distance. Panel C shows the combination of both but has a reduced Gaussian height and bias factor because of secondary structure unfolding.
(TIF)

S8 Fig Depiction of CVs used in different metadynamic simulations. The center of mass of the tryptophan residues was taken to bias the twisting of the β-hairpin motifs.
(TIF)

S1 File PCA 1. Projection of the first Eigenvector on the trajectory.
(MP4)

S2 File PCA 2. Projection of the second Eigenvector on the trajectory.
(MP4)

S3 File Supp comput. Input and parameter files used for structure elucidation and MD/metadynamics simulation.
(ZIP)

S4 File Readme. Detailed description of the compressed files in S4_supp_comput.zip.
(TXT)

S5 File Starting structure. NMR structure derived from a simulated annealing protocol implemented in Xplor-NIH.
(PDB)
