# Supplementary material for: Defining the mobility range of a hinge-type connection using molecular dynamics and metadynamics
Source: PLoS One. 2020 Apr 13;15(4):e0230962. doi: 10.1371/journal.pone.0230962 (PMC7153902; doi:10.1371/journal.pone.0230962)
Supplement: S1 Text — (DOCX) [file pone.0230962.s001.docx]

**Supporting information for**

**“Defining the mobility range of a hinge-like connection using molecular dynamics and metadynamics.”**

**S1 Text**

**Principal Component Analysis (PCA)**

We performed PCA on the whole data set of the classical molecular dynamics simulation to get an understanding of the most functional modes underlying the dynamic of the hinge peptide. Firstly, the positions of the backbone atoms were aligned, followed by the calculation and diagonalization of the covariance matrix. We opted for a depiction of the first 10 eigenvalues, since they account for 82% of the covariance of the peptide. The first and third eigenvectors are visualized as the twisting of the beta hairpins and account for 55%, while the opening and closing motion (largely resembled in Eigenvector 2 and 4) amounts to 11%.


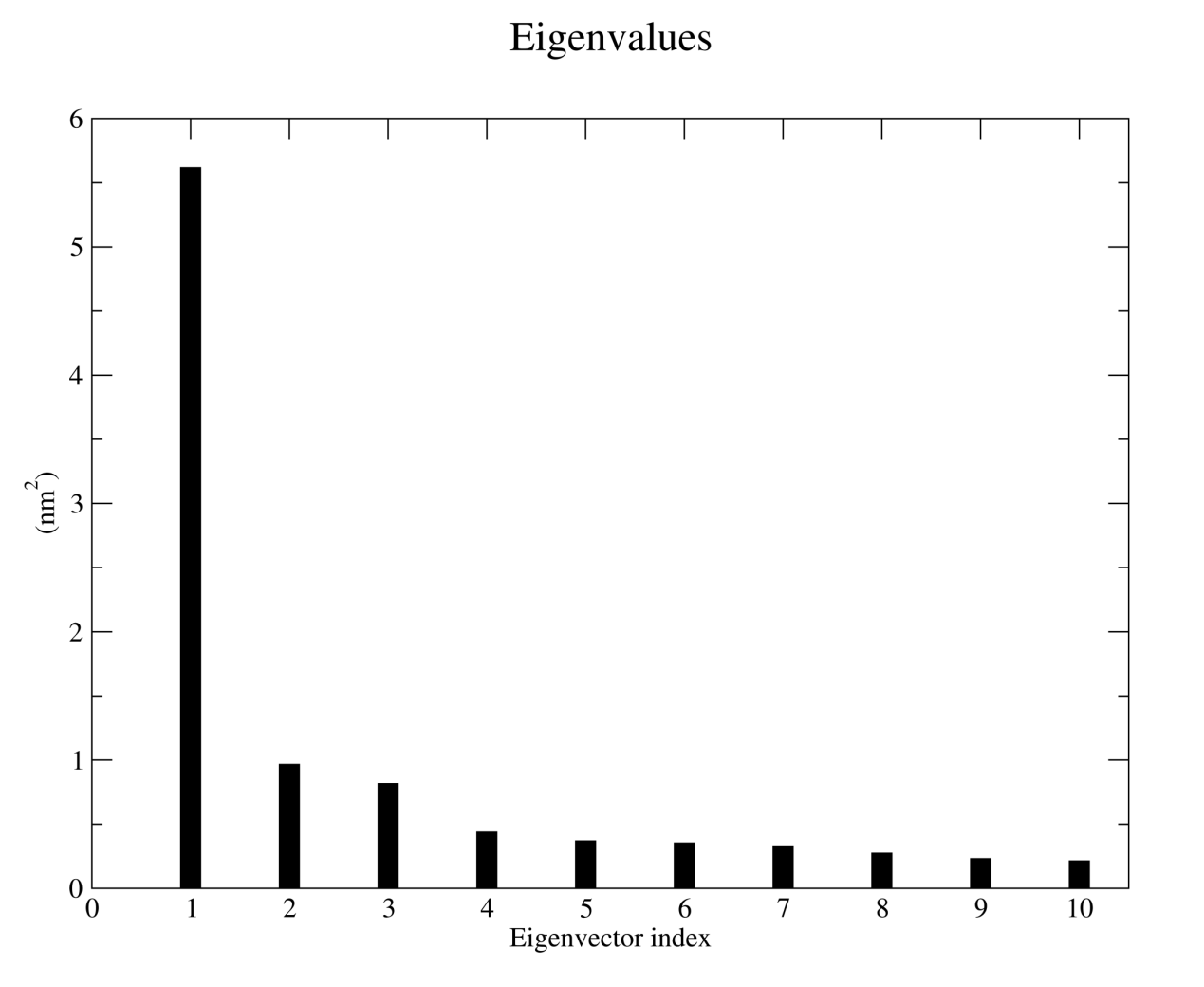


**Fig S1. Eigenvalues obtained after PCA performed on classical MD simulation.** Only the first 10 Eigenvectors are displayed since they account for 82% of the overall motion.

**NMR structure generation**

The NMR structure of the hinge peptide was determined by using the Xplor-NIH suite of programs (v. 2.47) (which can be obtained free of charge at https://nmr.cit.nih.gov/xplor-nih/). The eefx2 implicit solvent was used for simulated annealing calculations. The chains were built separately and joined by adding the respective disulfide bonds. Distance constraints were extracted from NOESY spectra with a mixing time of 300 ms. The signals were integrated in Topspin and the distance of the tryptophan aromatic protons to each other was used as the reference distance. The cross-peaks were, thus, divided into three categories: Weak, medium and strong. Since the system observed a higher flexibility, only a small set of 23 well-defined NOE restraints were generated. The analysis of coupling constants revealed preferred side-chain orientation. The dihedral restraint was allowed to deviate up to 15 or 30° in the case of the intra-disulfide bonds. This data set proved to be consistent in the deliverance of a structural motif without any NOE violations above 0.5 Å. An extended structure is generated in the protocol and used for the torsion angle dynamics simulated annealing. The sequence used was (CHWECRGCRLVC)_2_ with the N-terminus acetylated and the C-terminus deprotonated to closely resemble the experimental conditions. The calculation was performed for 5000 structures which took about three days on our system. The lowest energy conformers are resembled in Fig S2. The simulated annealing protocol in its modified form is supplied in the supp_comput.zip.


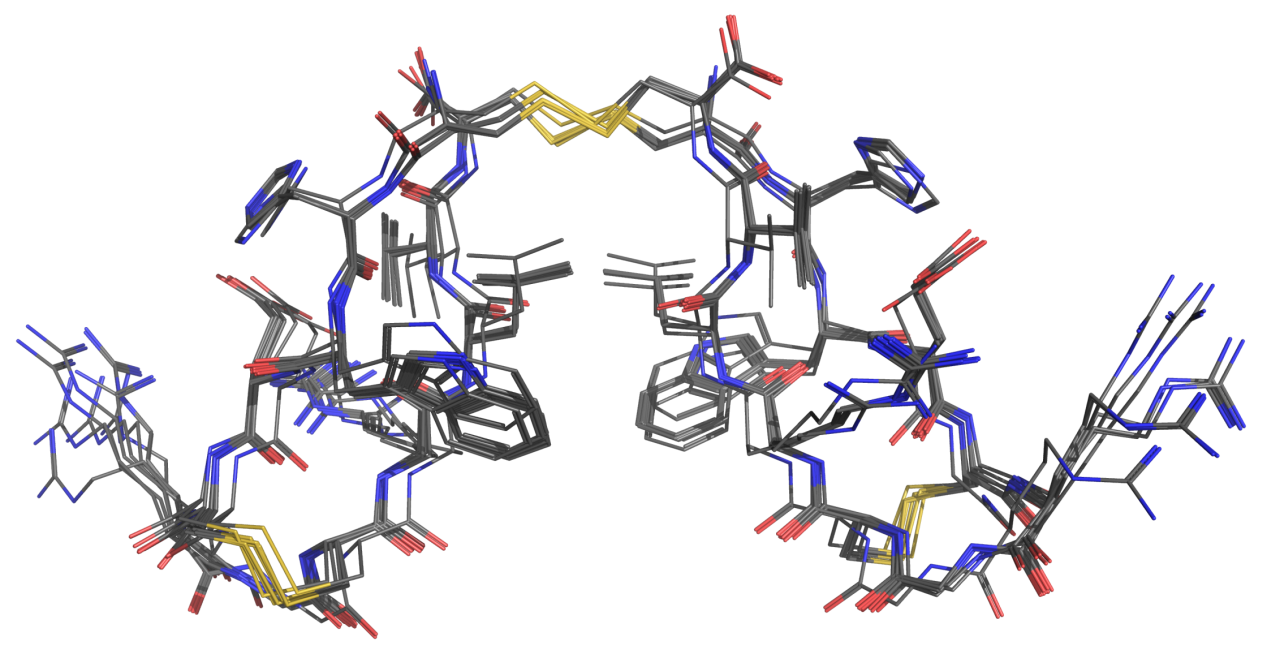


**Fig. S2. Lowest energy conformer assembly obtained after performing an NMR structure calculation using the simulated annealing protocol.**

**MD simulations**

All simulations were performed using the GROMACS 2018.4 suite patched with plumed 2.4. The GROMACS suite is available free of charge under the GNU Lesser General Public License at http://manual.gromacs.org/documentation/, while plumed is available at https://www.plumed.org/. The lowest energy structure originating from the NMR calculations was chosen for input preparation and processed using the pdb2gmx program set. The N-terminus was acetylated and the C-terminus deprotonated. Histidine and arginine side chains were also protonated. The chains were separated using the “ter” flag. Using the GROMACS suite, the system was solvated with ~6000 TIP3P water molecules in a dodecahedron box and a salt concentration of 0.15 M was added to neutralize the system. After evaluation, the OPLSAA/M force field was used, which is available at http://zarbi.chem.yale.edu/oplsaam.html. Energy minimization was performed for either 500,000 steps or until the maximum force reached a value below 50 kJ/mol/nm using a steepest-descent algorithm to remove steric clashes between the peptide and solvent. The next step included a 10-ns-long equilibration to allow the solvent to fully surround the peptide. The first equilibration was conducted under a NVT ensemble at 300 K using the modified Berendsen thermostat v-rescale with a coupling time step of 0.1 ps to stabilize the temperature of the system, followed by an 10-ns-long NPT equilibration to stabilize the pressure using the Berendsen barostat with a coupling time step of 2.0 ps. Particle-Mesh Ewald for the treatment of electrostatic interactions was employed with a short-range cut off of 1.0 nm. The Parrinello-Rahman barostat was used for the final unrestrained production run and the system subjected to a 2550-ns-long run at 300K. After the simulation had finished, corrections to periodic boundary were performed and rotational plus translational motions were removed for easier visualization in the Visual Molecular Dynamics package, which can be obtained at https://www.ks.uiuc.edu/Research/vmd/, and was also used to generate the graphical illustrations of the hinge peptide. The Root-Mean-Square Deviation (RMSD) and Root-Mean-Square Fluctuation (RMSF) were measured using implemented tools in the GROMACS package.

**Free energy estimation conversion**

One important measurement to perform after a metadynamics simulation is the convergence. One good estimation is the height of the Gaussian deployed during the simulation. As observed in Fig S3, the height of Gaussian slowly decreases. Only small energy values were deployed for the last 800 ns. Furthermore, if the simulation converged, there should still be crossing events even if no Gaussian is deposited. This is visible at 2300 ns, in which case, the distance of the tryptophan residues changes even though no Gaussian was previously deposited. An additional criterion for the conversion of the metadynamics can be the conversion of the free energy surface of the CV sampled by plumed. The overall shape of the FES does not change, apart from a constant offset during different simulation times. This can be another indication of a converged simulation. Fig S4, firstly, shows the FES over the whole simulation time frame, ranging from 50 – 2500 ns. Large changes of the shape can be observed from 50 – 1500 ns, after which, the surface stays consistent and only changes in the offset. To further highlight this behavior, the second part of Fig S4 shows the FES from 2000 – 2500 ns. Here, the changes in the FES are negligible. The two-dimensional FES analysis was performed, firstly, by calculating a histogram along the opening angle and W – W’ distance with the kernel density estimation implemented in plumed. Secondly, this data set was then converted to a free energy surface using a weighted histogram approach with a bin size of 425. To estimate the conversion of the FES during the simulation, this approach was performed at specific time intervals (after 500, 1000, 2000 and 2550 ns) and is depicted in Fig S5. Additionally, we wanted to measure the effect of the unconverged trajectory on the FES. Therefore, the bottom of Fig S5 shows the truncated FES from 500 – 2500 ns, which closely resembles the full FES.


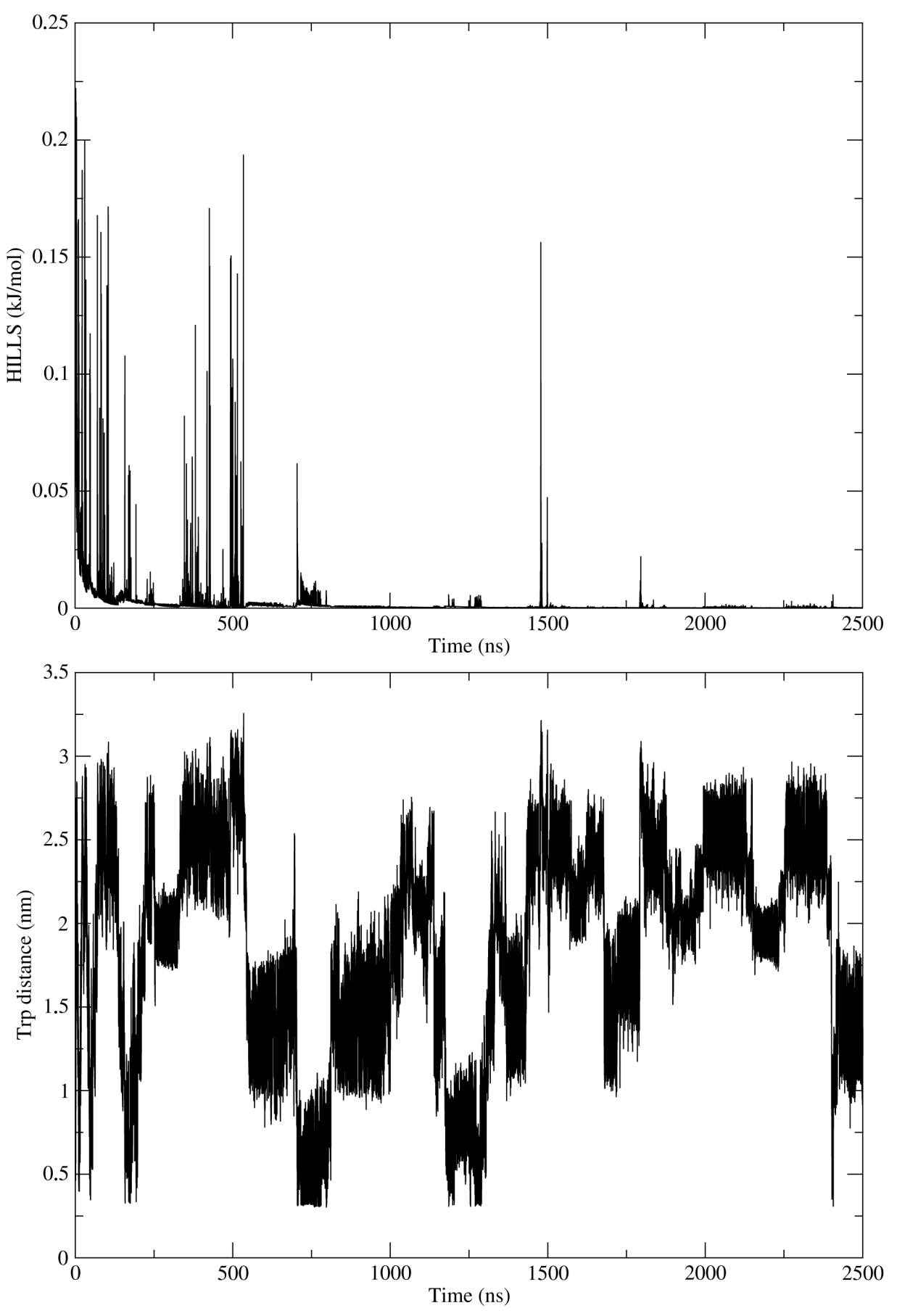


**Fig S3. Metadynamics conversion estimation.** On the top, the HILLS file generated during the metadynamics simulation shows a constant decline of the deposited Gaussian height. On the bottom, tryptophan distance used as the collective variable shows fluctuations even after no big Gaussian is deployed, signaling a conversion of the simulation.


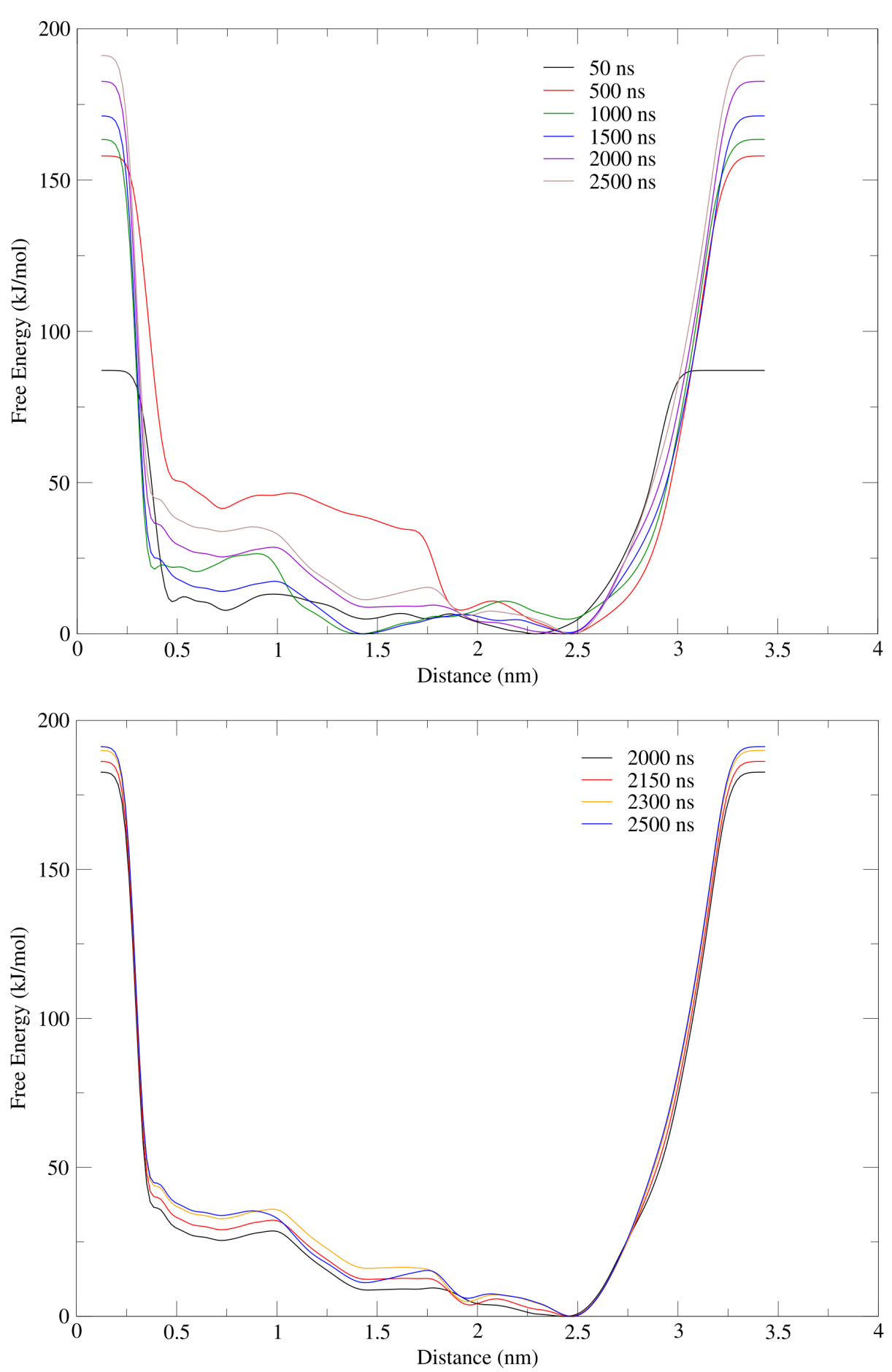


**Fig S4. Free energy surface at different time points.** On the top, the graph depicts the FES of the biased parameter during the metadynamics (W – W’ distance) at different time points over the whole simulation. The bottom illustration highlights the resemblance between the free energy profiles at different time steps during the end of the simulation.


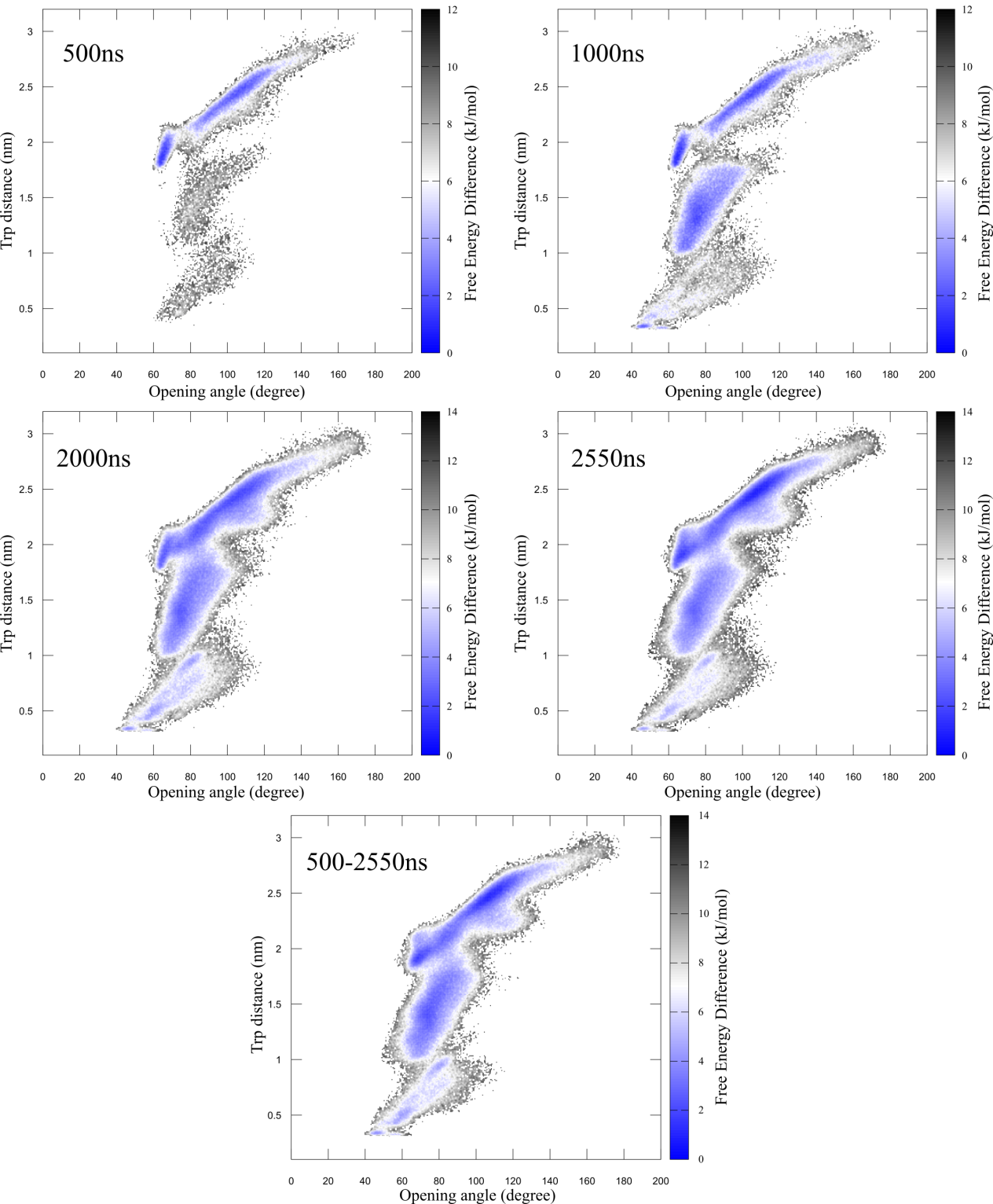


**Fig S5. Two-dimensional free energy surface evolution.** Depiction of the two-dimensional FES at different time points. Large changes can be observed between 500 and 1000 ns but after 2000 ns, the surface changes are only negligibly small. The bottom illustration shows the FES omitting the first 500 ns of the metadynamics simulation.

**Error estimation of the most relevant free energy basins**

We performed block analysis to estimate error of the most relevant free energy basins obtained during the metadynamics. Firstly the associated weights for each conformation were calculated using a umbrella-sampling reweighting approach, followed by the block analysis, which yields the average free energy and the error for the specific block size. The final free energy profile along the CV combined with the free energy error estimation is depicted in Fig S6.


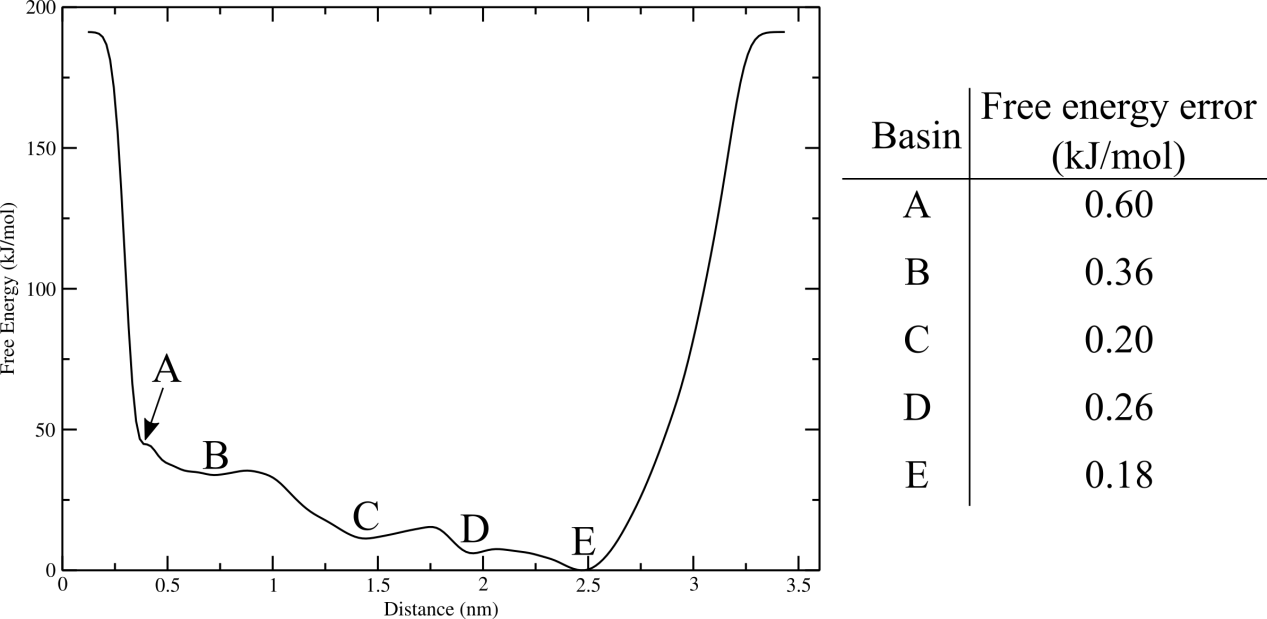


**Fig S6. Free energy estimate profile in combination with error estimation.** The graph shows the final FES obtained at the end of the simulation in analogy to Fig S4. The main basins are labeled and the table on the right shows an estimation of the errors according to each basin.

**Examination of different CVs**

As mentioned in the main article, we chose the tryptophan distance as the only CV. This choice was motivated by performing PCA and identifying the twisting motion as the dominant mode. Nonetheless, we performed metadynamics using the opening angle as a CV and a combination of those two parameters. We had to lower the Gaussian height and the bias factor for the opening angle, since the initial parameters were unfolding the hairpin structure. The combination of the two CVs led to a quick unfolding and resulted in a drastic decrease of the conformational space defined.


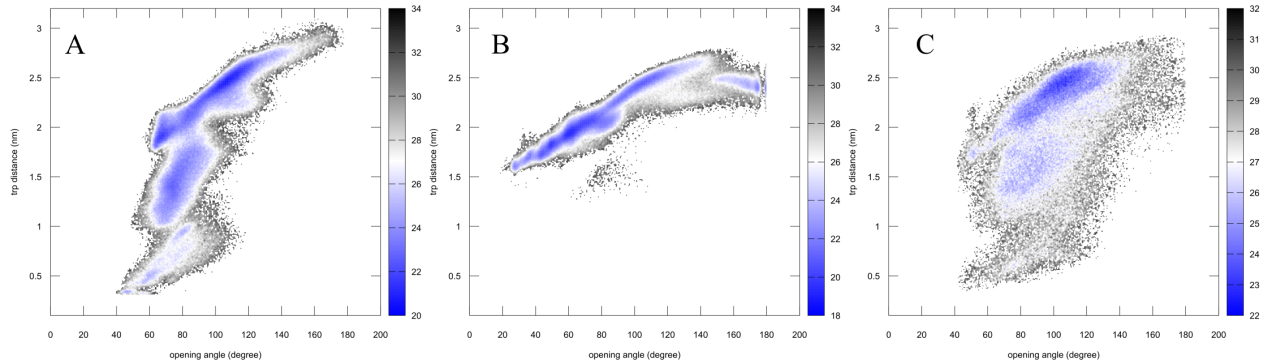


**Fig S7. Free energy surface representations of different metadynamics.** A shows the FES of the metadynamics simulation using the tryptophan distance as a CV, while B (opening angle is biased) shows a narrow valley extended in the opening angle but restricted in the trp distance. Panel C shows the combination of both but has a reduced Gaussian height and bias factor because of secondary structure unfolding.

**Description of the tryptophan distance and opening angle**

As shown in Fig S4, we used two different CVs, namely, the trp distance derived from the center of mass (COM) from the trp side chains. The second CV was the opening angle constituted from the COM of the carbon alpha atoms of the intra-disulfide and the COM of the inter-disulfide cluster, as depicted in Fig S5.


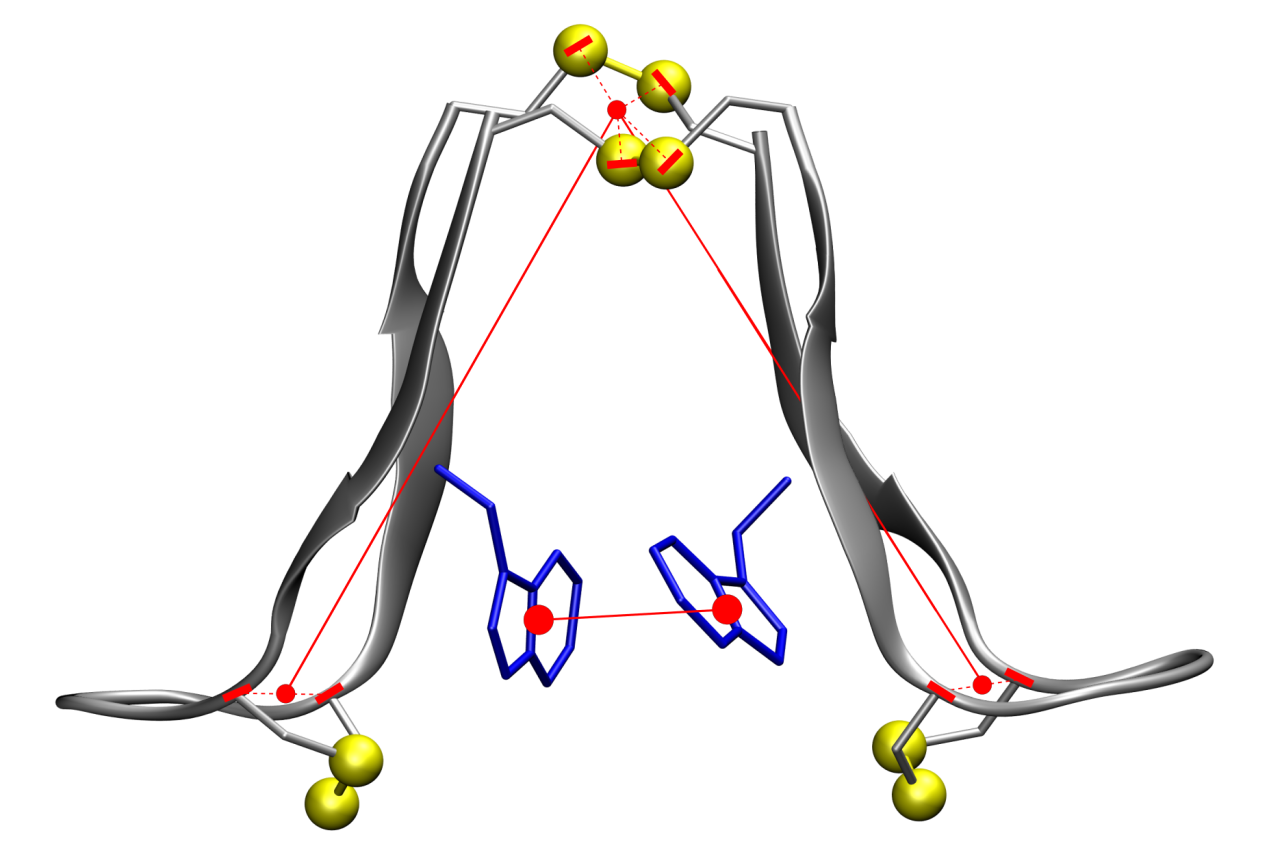


**Fig. S8. Depiction of CVs used in different metadynamic simulations.** The center of mass of the tryptophan residues was taken to bias the twisting of the beta-hairpin motifs.

**Visualization of the principal component analysis**

After performing PCA on the whole dataset of the standard MD simulation. Subsequently, the motion was displayed along the first two Eigenvectors and illustrated as videos alongside the supplementary data named S2_PCA_1.mp4 and S3_PCA_2.mp4.
