# Supplementary figures and images for: Defining the mobility range of a hinge-type connection using molecular dynamics and metadynamics
Source: PLoS One. 2020 Apr 13;15(4):e0230962. doi: 10.1371/journal.pone.0230962 (PMC7153902; doi:10.1371/journal.pone.0230962)

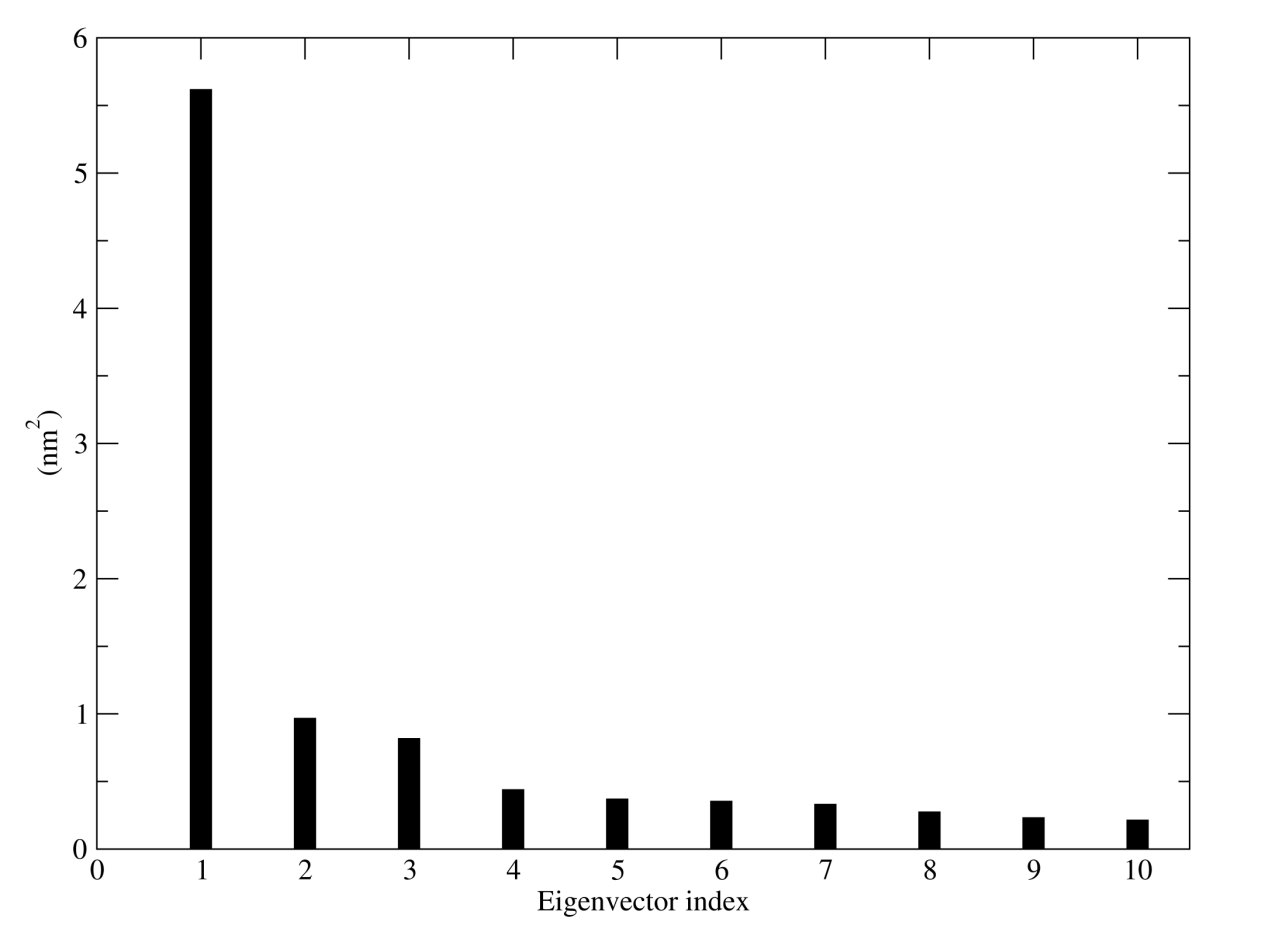

Supplement: S1 Fig — Only the first 10 Eigenvectors are displayed since they account for 82% of the overall motion. (TIF) [file pone.0230962.s002.tif]

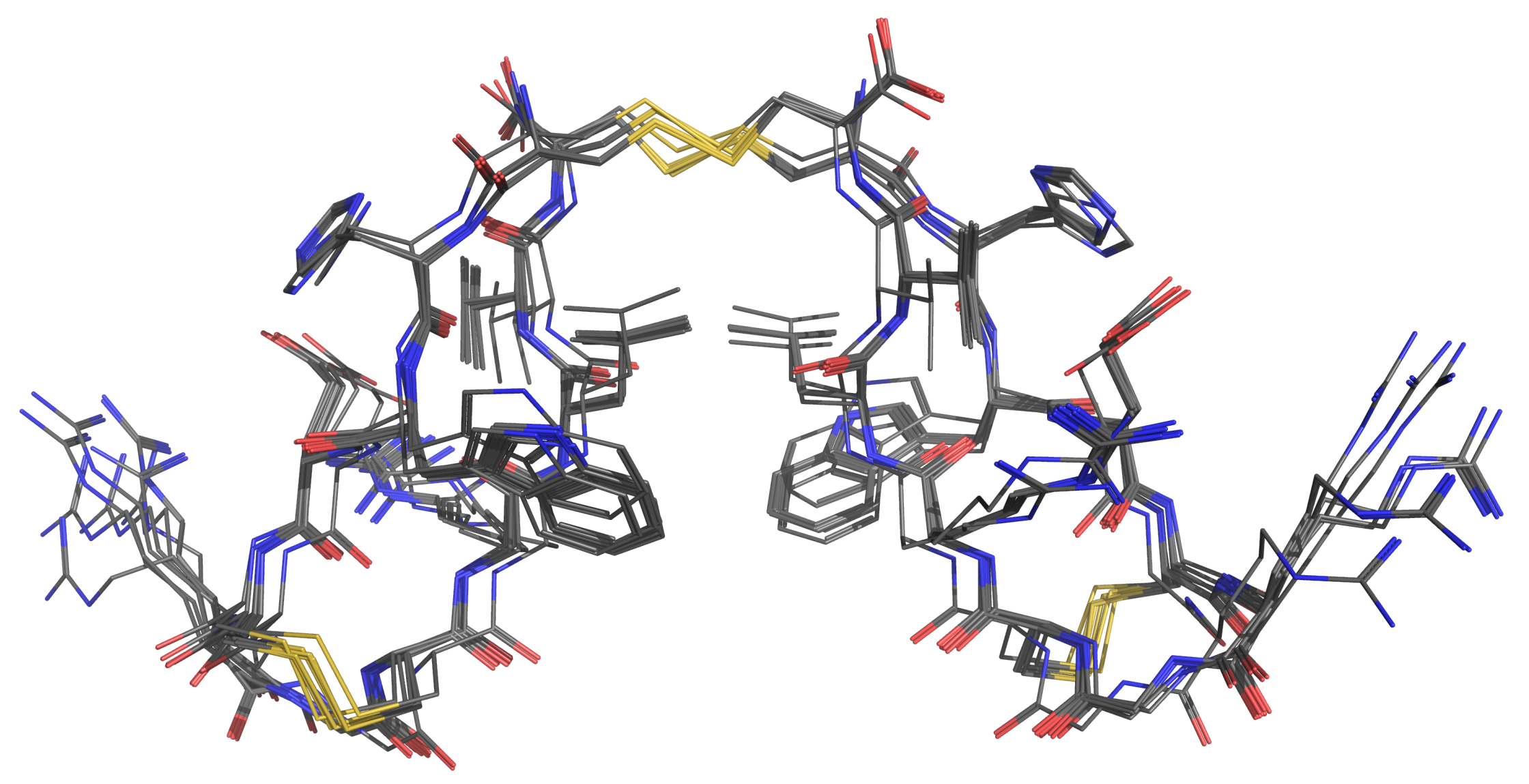

Supplement: S2 Fig — (TIF) [file pone.0230962.s003.tif]

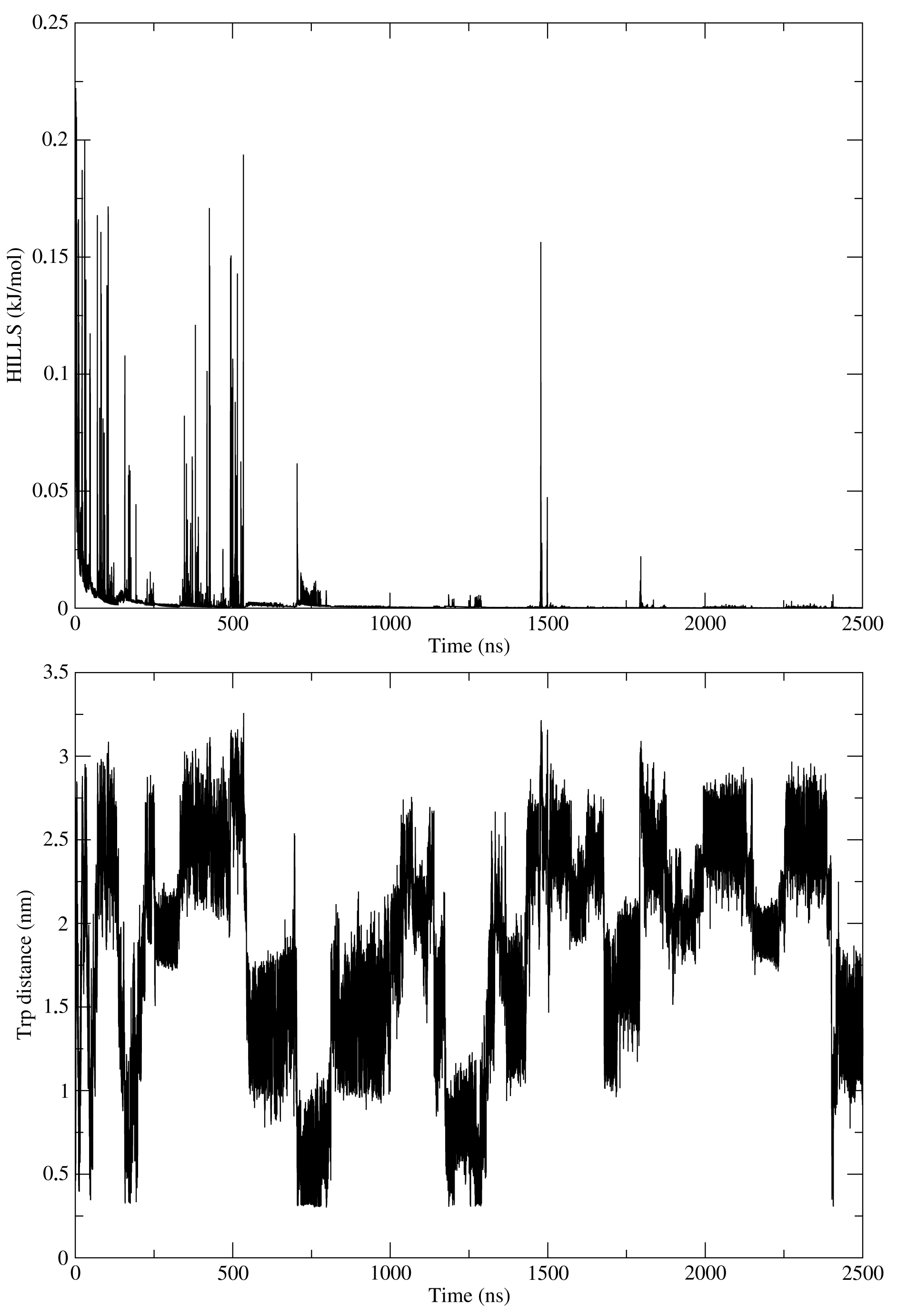

Supplement: S3 Fig — On the top, the HILLS file generated during the metadynamics simulation shows a constant decline of the deposited Gaussian height. On the bottom, tryptophan distance used as the collective variable shows fluctuations even after no big Gaussian is deployed, signaling a conversion of the simulation. (TIF) [file pone.0230962.s004.tif]

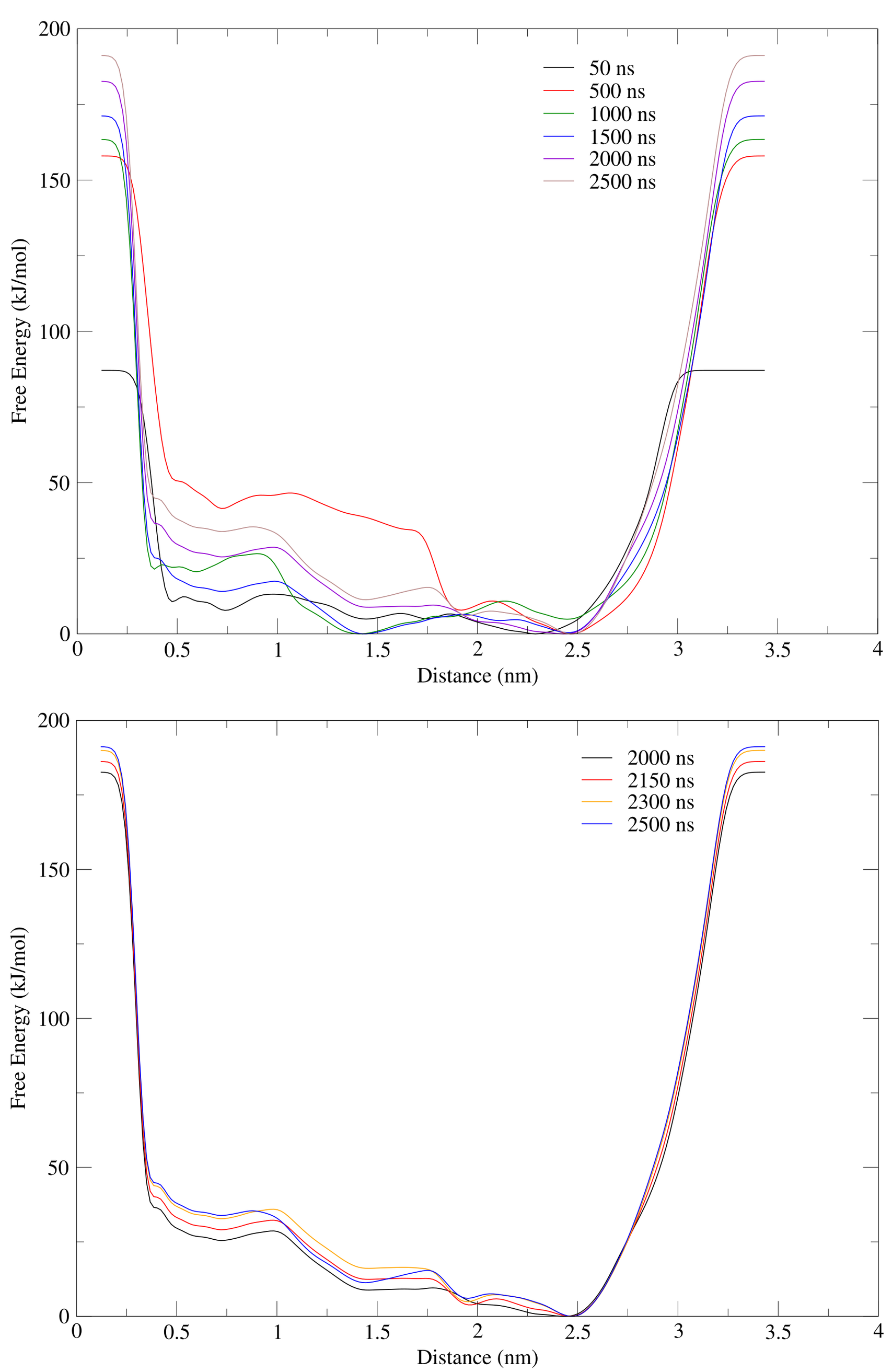

Supplement: S4 Fig — On the top, the graph depicts the FES of the biased parameter during the metadynamics (W–W’ distance) at different time points over the whole simulation. The bottom illustration highlights the resemblance between the free energy profiles at different time steps during the end of the simulation. (TIF) [file pone.0230962.s005.tif]

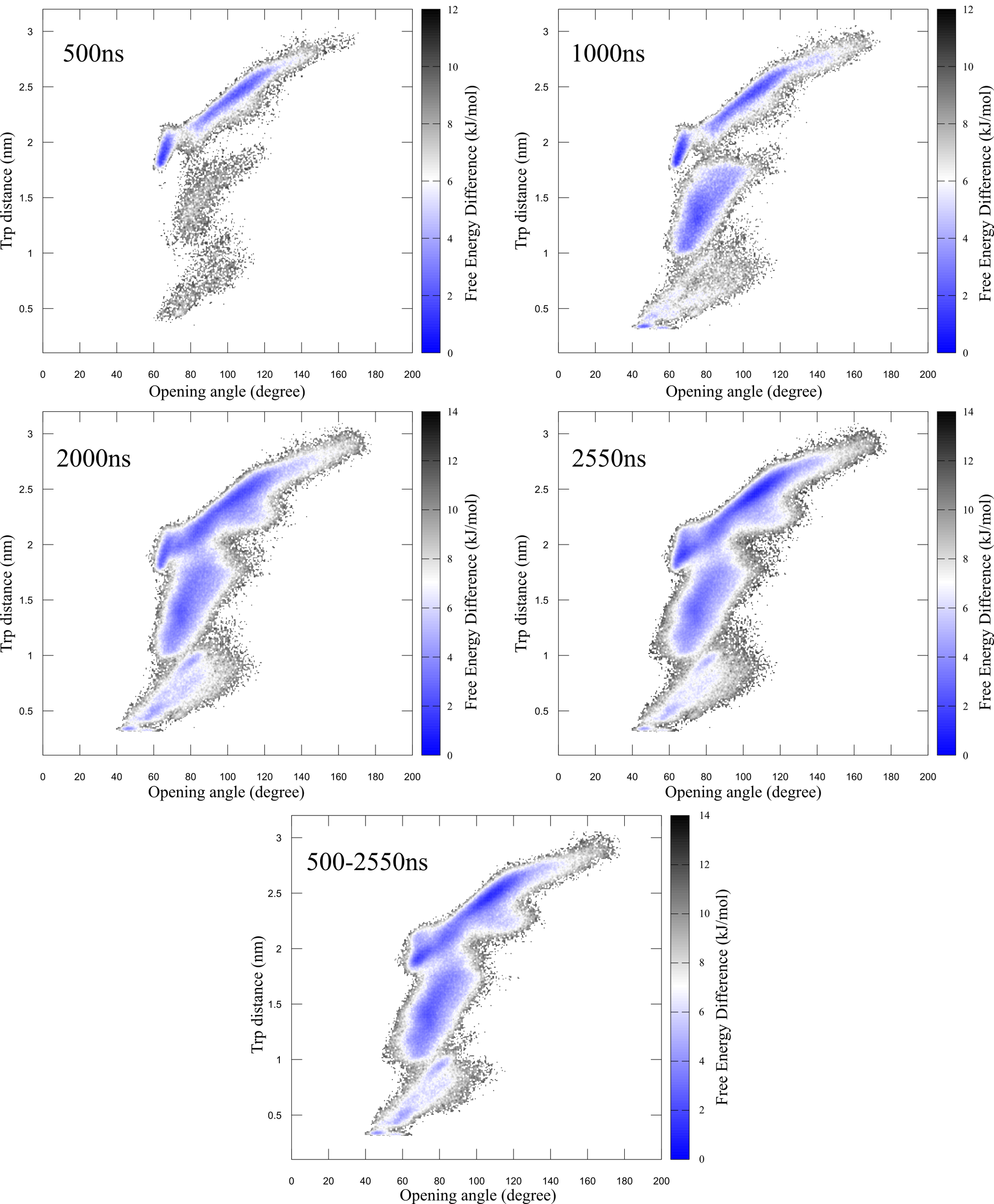

Supplement: S5 Fig — Depiction of the two-dimensional FES at different time points. Large changes can be observed between 500 and 1000 ns but after 2000 ns, the surface changes are only negligibly small. The bottom illustration shows the FES omitting the first 500 ns of the metadynamics simulation. (TIF) [file pone.0230962.s006.tif]

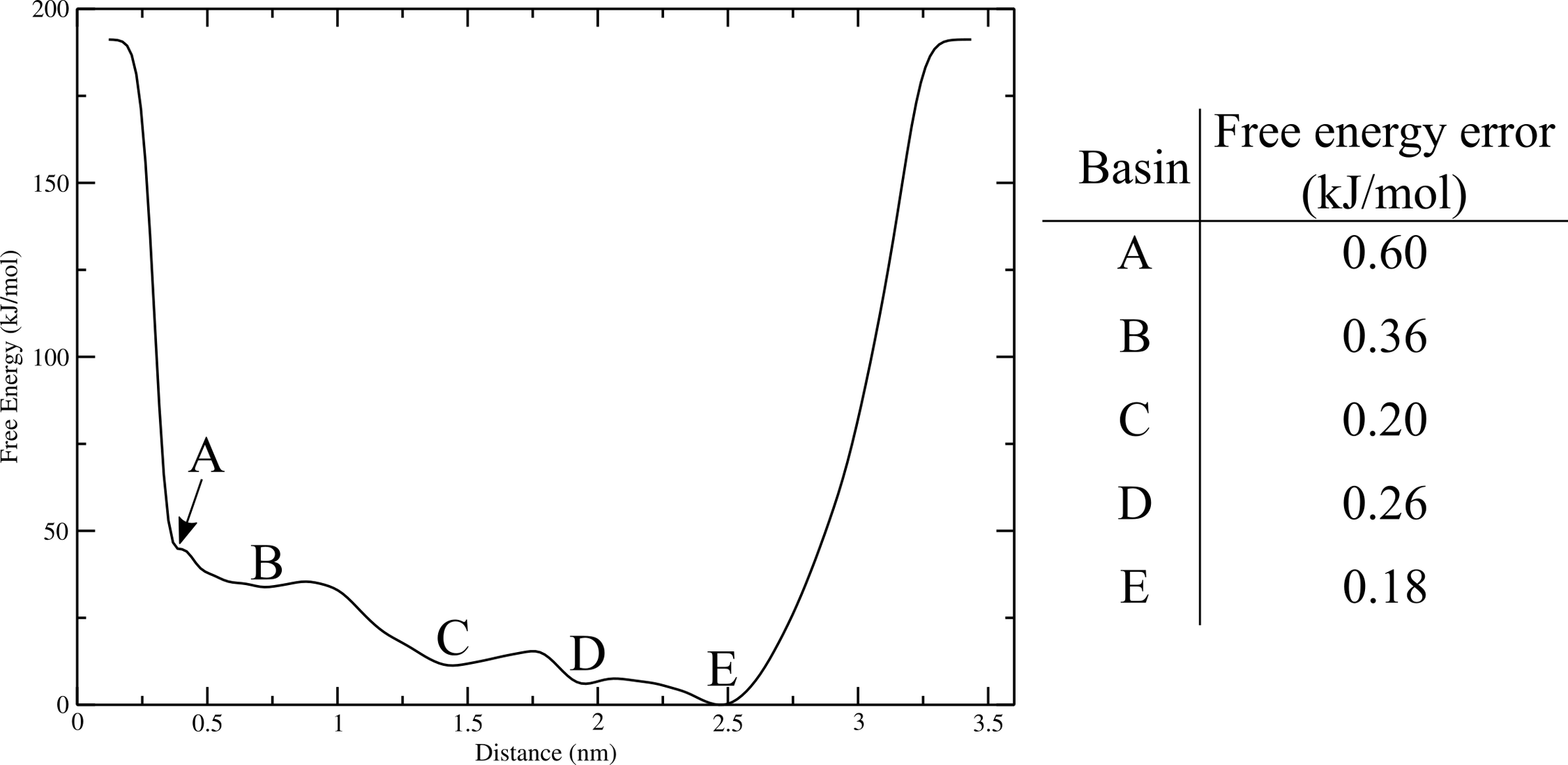

Supplement: S6 Fig — The graph shows the final FES obtained at the end of the simulation in analogy to S4 Fig. The main basins are labeled and the table on the right shows an estimation of the errors according to each basin. (TIF) [file pone.0230962.s007.tif]

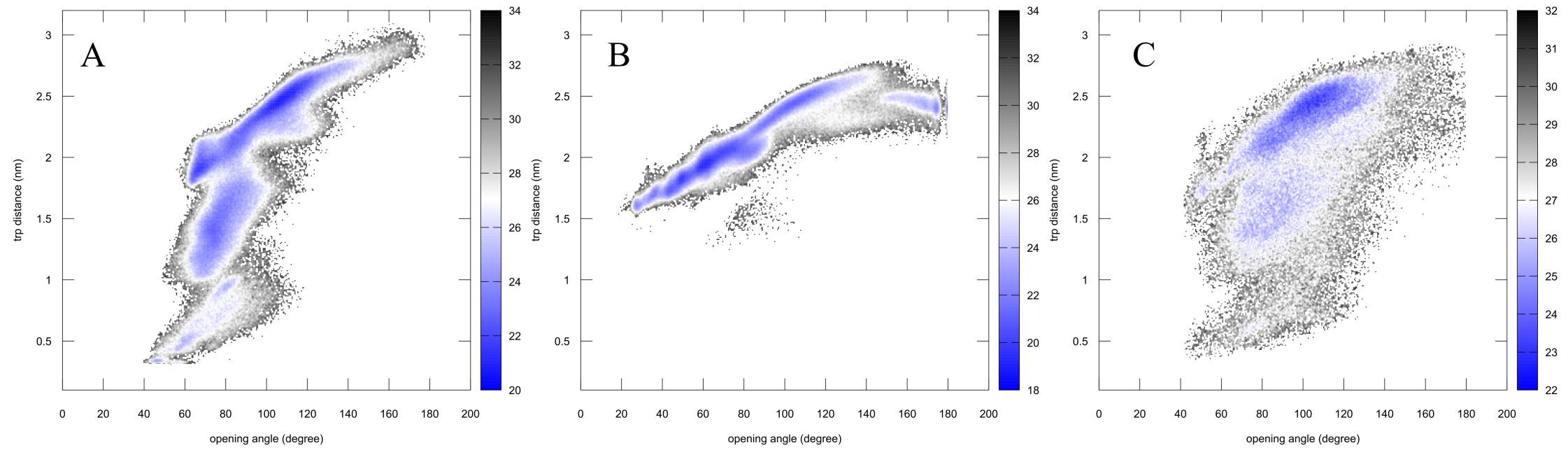

Supplement: S7 Fig — A shows the FES of the metadynamics simulation using the tryptophan distance as a CV, while B (opening angle is biased) shows a narrow valley extended in the opening angle but restricted in the trp distance. Panel C shows the combination of both but has a reduced Gaussian height and bias factor because of secondary structure unfolding. (TIF) [file pone.0230962.s008.tif]

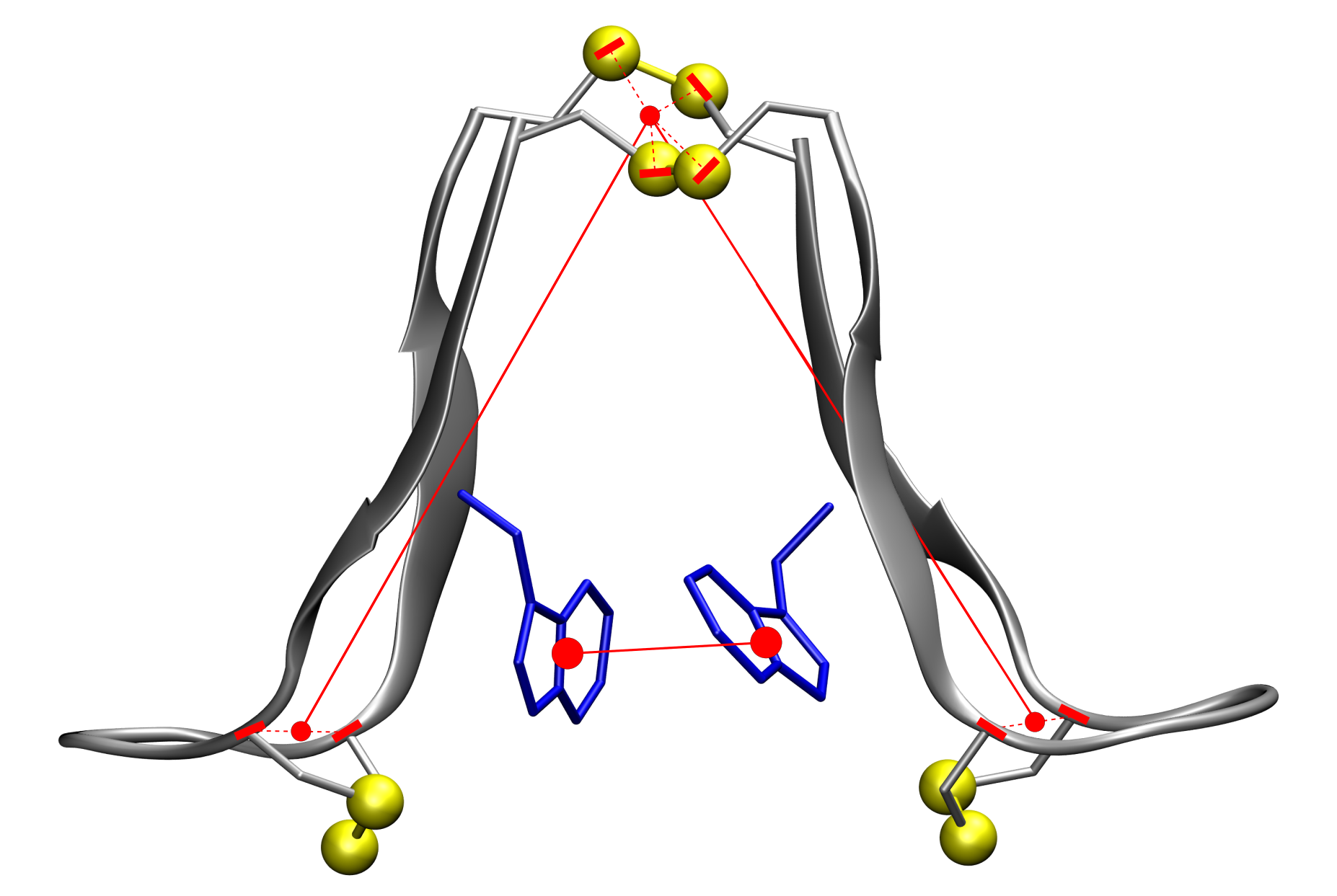

Supplement: S8 Fig — The center of mass of the tryptophan residues was taken to bias the twisting of the β-hairpin motifs. (TIF) [file pone.0230962.s009.tif]
